# Supplementary material for: Generalized logistic growth modeling of the COVID-19 outbreak: comparing the dynamics in the 29 provinces in China and in the rest of the world
Source: Nonlinear Dyn. 2020 Aug 19;101(3):1561–81. doi: 10.1007/s11071-020-05862-6 (PMC7437112; doi:10.1007/s11071-020-05862-6)
Supplement: Supplementary file 1 — Supplementary material 1 (PDF 2565 kb) [file 11071_2020_5862_MOESM1_ESM.pdf]

Supplementary Material

This supplementary material provides detailed fitting statistics and figures for each of the 29 Chinese provinces.

Table S1 provides useful statistics for each province and the values of the fitted parameters of the generalized Richards model, logistic growth model and the exponential decay exponent of the growth rate.

The figures afterwards present the total number of confirmed cases (upper panel), the daily number of new confirmed cases (middle panel), and the daily growth rate of confirmed cases (lower panel) in each of 29 provinces in the mainland China excluding Hubei and Tibet until March 1st, 2020. The empirical data is marked by the empty circles. The blue and red lines in the upper, middle and lower left panels show the fits with the Logistic Growth Model and Generalized Richards Model (GRM) respectively. For the GRM, we also show the fits using data ending 20, 15, 10, 5 days earlier than March 1st, 2020, as lighter red lines in the upper and middle panel. This demonstrates the consistency and robustness of the fits. The lower left panel shows the daily growth rate of the confirmed cases in log scale against time. The green straight lines show the linear regression of the logarithm of the growth rate as a function of time. The lower right panel is the daily growth rate of the confirmed cases in linear scale against the cumulative number of confirmed cases. The red and green lines are the linear fits for the period of Jan 19 to Feb 1, and the period of Feb 2 to Mar 1, respectively.

Table S1: Summary statistics for 29 provinces in mainland China (excluding Hubei and Tibet), as of Feb 29, 2020. The values of the fitted parameters of the generalized Richards model, logistic growth model and the exponential decay exponent are reported.

	Cumulative number of cases	Population density (2018, per km2)	Population (2018, million)	Date of 1st reported case	Generalized Richards Model					Logistic Growth Model				Exponential decay of the growth rate			# of days between 1st reported date and the peak of daily
					K	r	p	a	R-squared	K	C0	r	R-squared	a	95% Confidence Interval	R-squared	
Guangdong	1352	481	111.7	19-Jan-20	1322	1.00	0.84	0.74	90.7%	1344	24.3	0.29	91.1%	-0.159	(-0.139, -0.179)	92.7%	13
Henan	1272	553	95.6	21-Jan-20	1266	2.42	0.61	1.88	74.4%	1302	37.6	0.28	75.0%	-0.186	(-0.161, -0.210)	92.8%	11
Zhejiang	1242	460	56.6	20-Jan-20	1190	1.30	1.00	0.19	82.3%	1220	37.0	0.31	80.8%	-0.223	(-0.201, -0.244)	95.0%	10
Hunan	1018	304	68.6	21-Jan-20	1043	2.94	0.56	1.68	91.8%	1084	50.9	0.25	91.4%	-0.166	(-0.154, -0.178)	97.4%	12
Anhui	990	429	62.5	20-Jan-20	987	3.78	0.47	3.48	91.8%	1045	24.0	0.25	92.1%	-0.167	(-0.149, -0.186)	90.4%	15
Shandong	960	579	100.1	20-Jan-20	553	3.69	0.39	4.83	69.8%	604	27.2	0.22	67.6%	-0.163	(-0.138, -0.189)	90.4%	17
Jiangxi	935	247	46.2	20-Jan-20	966	0.99	0.82	0.75	89.0%	980	19.8	0.27	88.4%	-0.173	(-0.156, -0.190)	94.8%	15
Jiangsu	631	742	80.3	22-Jan-20	655	3.40	0.45	2.45	93.6%	697	43.9	0.22	93.0%	-0.149	(-0.139, -0.158)	97.4%	10
Chongqing	576	374	30.5	21-Jan-20	584	3.90	0.41	2.18	80.6%	633	61.7	0.20	80.5%	-0.142	(-0.121, -0.163)	90.0%	11
Sichuan	539	172	83.0	20-Jan-20	564	11.73	0.87	0.02	87.0%	597	51.8	0.18	82.7%	-0.145	(-0.130, -0.159)	94.9%	11
Heilongjiang	481	81	37.9	21-Jan-20	481	0.63	1.00	0.34	73.9%	494	7.9	0.26	73.7%	-0.118	(-0.091, -0.145)	67.6%	14
Beijing	426	1323	21.7	20-Jan-20	401	0.98	0.68	1.49	77.8%	413	19.4	0.23	78.4%	-0.156	(-0.122, -0.189)	84.1%	11
Shanghai	342	3814	24.2	20-Jan-20	330	1.49	0.59	1.88	86.7%	345	14.0	0.26	88.1%	-0.160	(-0.138, -0.182)	87.9%	11
Hebei	318	355	75.2	22-Jan-20	310	3.16	0.31	11.82	72.0%	350	22.2	0.19	65.4%	-0.130	(-0.099, -0.161)	79.9%	16
Fujian	296	285	39.1	22-Jan-20	302	39.51	0.91	0.01	91.0%	315	27.9	0.26	85.8%	-0.200	(-0.180, -0.220)	95.2%	7
Guangxi	252	190	48.9	20-Jan-20	251	4.64	0.22	3.92	68.4%	292	28.1	0.18	68.5%	-0.144	(-0.102, -0.186)	76.2%	11
Shaanxi	245	185	38.4	22-Jan-20	249	1.11	0.68	1.02	70.0%	258	20.4	0.24	70.0%	-0.171	(-0.140, -0.201)	87.4%	10
Yunnan	174	109	48.0	20-Jan-20	169	11.86	1.00	0.02	63.7%	161	6.2	0.33	57.2%	-0.195	(-0.146, -0.245)	75.5%	9
Hainan	168	224	9.3	20-Jan-20	168	2.49	0.27	8.07	65.2%	189	11.9	0.19	60.1%	-0.084	(-0.038, -0.129)	49.2%	16
Guizhou	146	200	35.8	20-Jan-20	150	0.66	0.61	8.39	75.0%	156	1.7	0.27	72.1%	-0.150	(-0.112, -0.188)	78.5%	20
Tianjin	136	1306	15.6	21-Jan-20	138	0.86	0.53	2.62	45.2%	146	8.4	0.19	45.4%	-0.111	(-0.057, -0.164)	44.7%	14
Shanxi	133	212	37.0	21-Jan-20	130	1.72	0.38	7.44	84.9%	142	5.8	0.26	83.2%	-0.193	(-0.170, -0.217)	91.7%	13
Liaoning	125	291	43.7	22-Jan-20	124	2.02	0.35	2.92	62.0%	137	15.3	0.22	62.0%	-0.132	(-0.091, -0.173)	74.1%	7
Jilin	119	151	27.2	20-Jan-20	87	2.18	0.22	25.81	66.0%	103	10.3	0.23	63.1%	-0.187	(-0.139, -0.236)	78.0%	14
Gansu	93	57	26.3	23-Jan-20	87	0.32	1.00	1.41	72.8%	83	0.1	0.44	74.6%	-0.158	(-0.117, -0.199)	77.4%	9
Xinjiang	76	13	24.4	23-Jan-20	78	0.92	0.38	11.42	68.7%	83	4.3	0.20	60.1%	-0.087	(-0.063, -0.110)	59.7%	18
Neimenggu/Inner Mongolia	75	20	25.3	23-Jan-20	77	1.56	0.28	2.94	50.9%	88	11.7	0.18	51.2%	-0.135	(-0.092, -0.179)	67.9%	11
Ningxia	75	85	6.8	20-Jan-20	71	1.27	0.27	88.28	55.7%	84	6.2	0.17	41.3%	-0.102	(-0.049, -0.155)	50.6%	22
Qinghai	18	7	6.0	24-Jan-20	18	1.20	0.13	100.00	50.9%	24	4.0	0.27	39.7%	-0.227	(-0.092, -0.362)	71.0%	8
**China exclude Hubei**	**13213**	**141**	**1325.7**	**19-Jan-20**	**12919**	**4.87**	**0.63**	**1.42**	**97.3%**	**13234**	**382.8**	**0.24**	**96.6%**	**-0.157**	**(-0.164, -0.150)**	**99.2%**	**14**

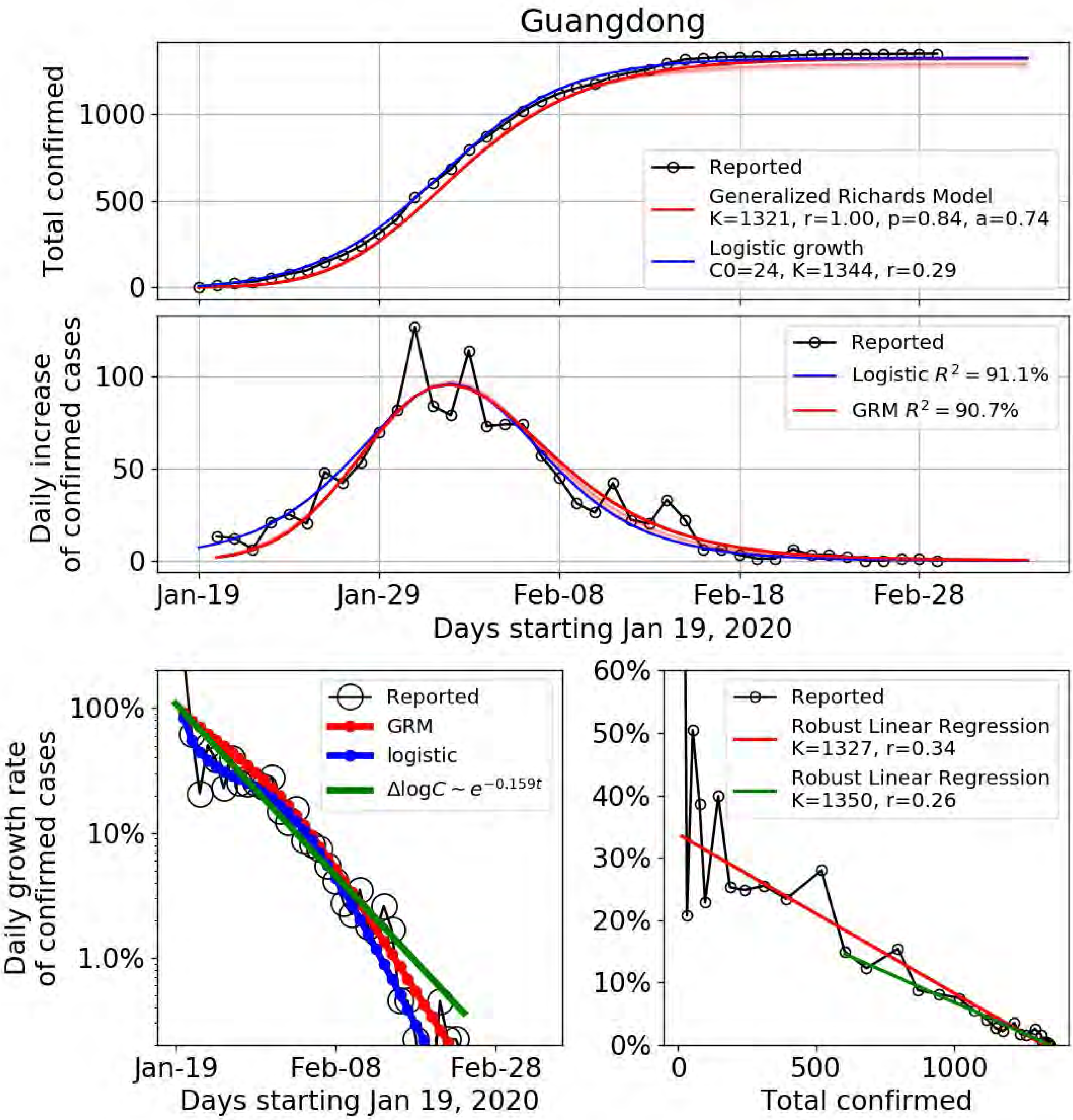

Guangdong
Total confirmed
Reported
Generalized Richards Model
K=1321, r=1.00, p=0.84, a=0.74
Logistic growth
C0=24, K=1344, r=0.29
Daily increase of confirmed cases
Logistic $R^2 = 91.1\%$
GRM $R^2 = 90.7\%$
Jan-19
Jan-29
Feb-08
Feb-18
Feb-28
Days starting Jan 19, 2020
Daily growth rate of confirmed cases
GRM
logistic
$\Delta \log C \sim e^{-0.159t}$
Robust Linear Regression
K=1327, r=0.34
Robust Linear Regression
K=1350, r=0.26
Total confirmed

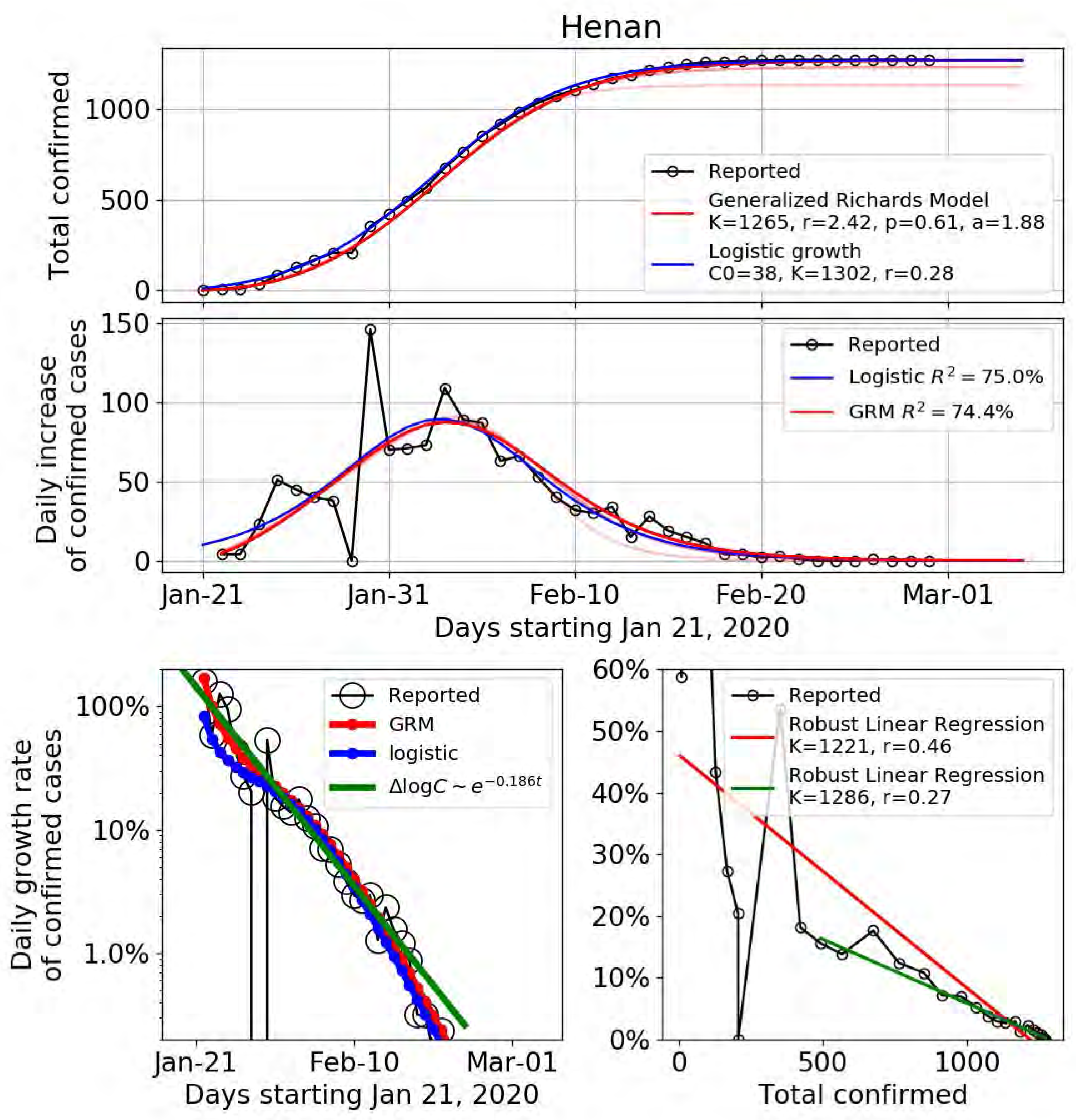

Henan
Total confirmed
Reported
Generalized Richards Model
K=1265, r=2.42, p=0.61, a=1.88
Logistic growth
C0=38, K=1302, r=0.28
Daily increase of confirmed cases
Logistic $R^2 = 75.0\%$
GRM $R^2 = 74.4\%$
Jan-21
Jan-31
Feb-10
Feb-20
Mar-01
Days starting Jan 21, 2020
Daily growth rate of confirmed cases
GRM
logistic
$\Delta \log C \sim e^{-0.186t}$
Robust Linear Regression
K=1221, r=0.46
Robust Linear Regression
K=1286, r=0.27
Total confirmed

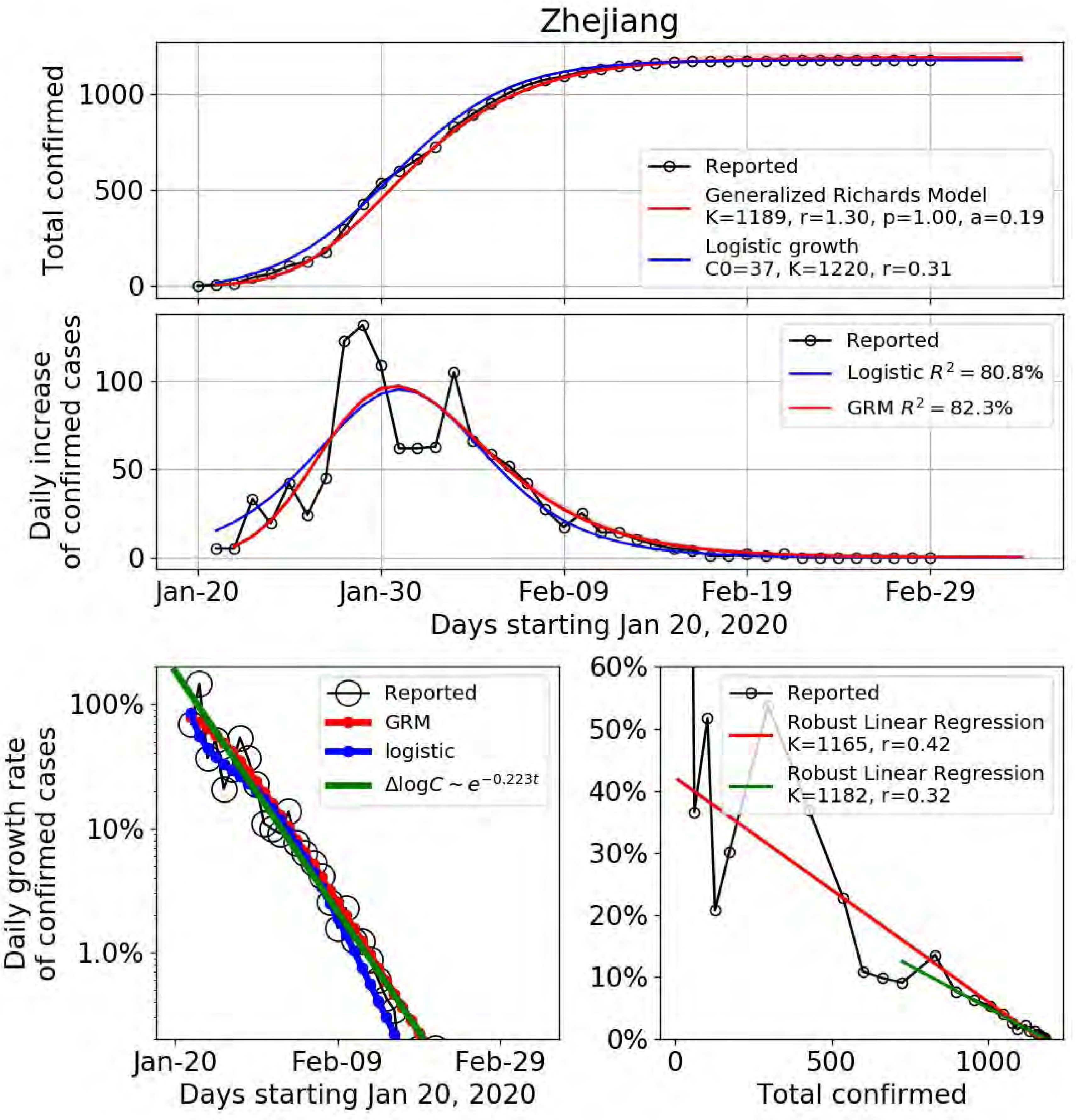
Zhejiang
Total confirmed
Reported
Generalized Richards Model
K=1189, r=1.30, p=1.00, a=0.19
Logistic growth
C0=37, K=1220, r=0.31
Daily increase of confirmed cases
Reported
Logistic $R^2 = 80.8\%$
GRM $R^2 = 82.3\%$
Jan-20
Jan-30
Feb-09
Feb-19
Feb-29
Days starting Jan 20, 2020
Daily growth rate of confirmed cases
Reported
GRM
logistic
$\Delta \log C \sim e^{-0.223t}$
Days starting Jan 20, 2020
Reported
Robust Linear Regression
K=1165, r=0.42
Robust Linear Regression
K=1182, r=0.32
Total confirmed

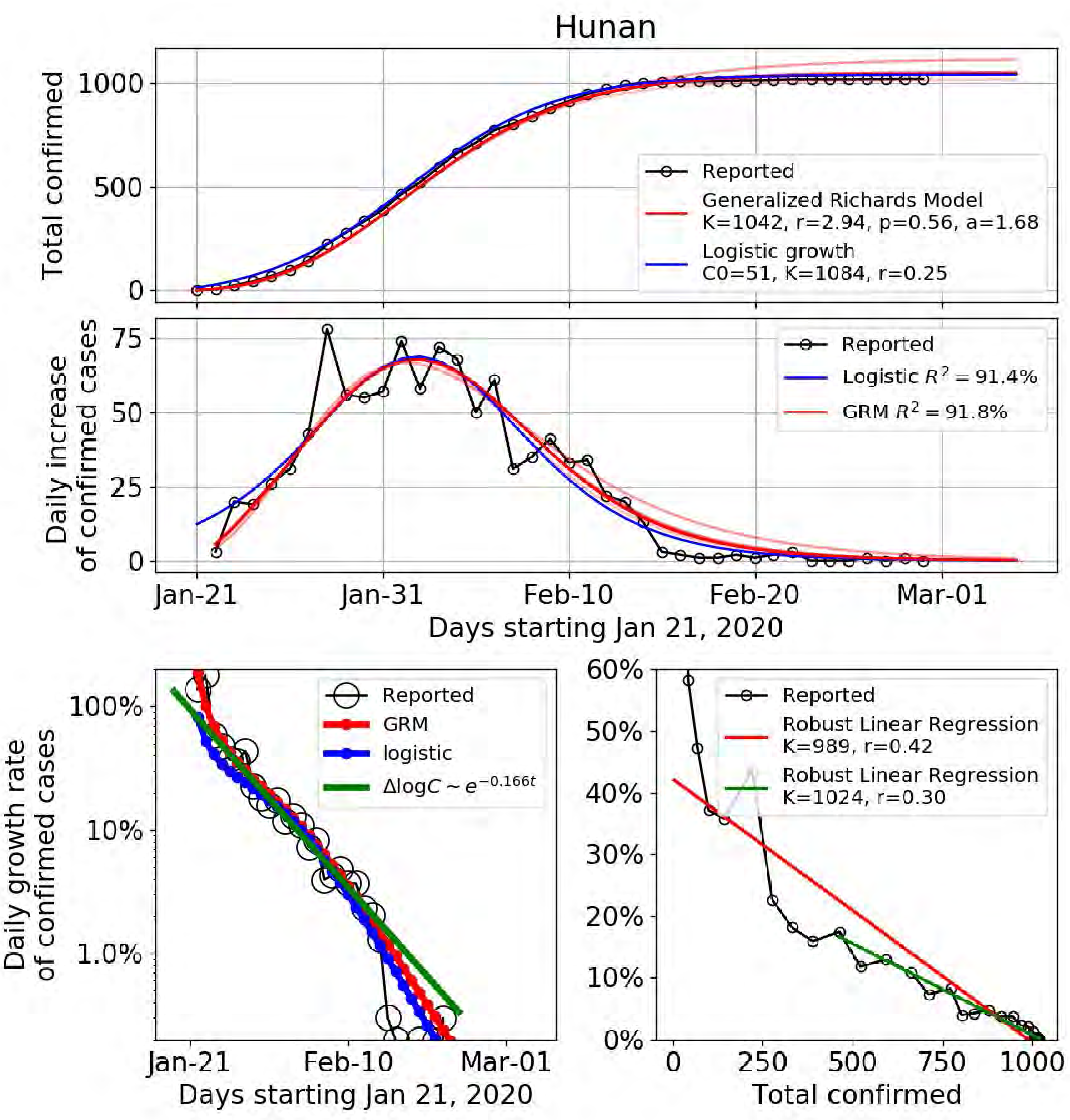
Hunan
Total confirmed
Reported
Generalized Richards Model
K=1042, r=2.94, p=0.56, a=1.68
Logistic growth
C0=51, K=1084, r=0.25
Daily increase of confirmed cases
Reported
Logistic $R^2 = 91.4\%$
GRM $R^2 = 91.8\%$
Jan-21
Jan-31
Feb-10
Feb-20
Mar-01
Days starting Jan 21, 2020
Daily growth rate of confirmed cases
Reported
GRM
logistic
$\Delta \log C \sim e^{-0.166t}$
Days starting Jan 21, 2020
Reported
Robust Linear Regression
K=989, r=0.42
Robust Linear Regression
K=1024, r=0.30
Total confirmed

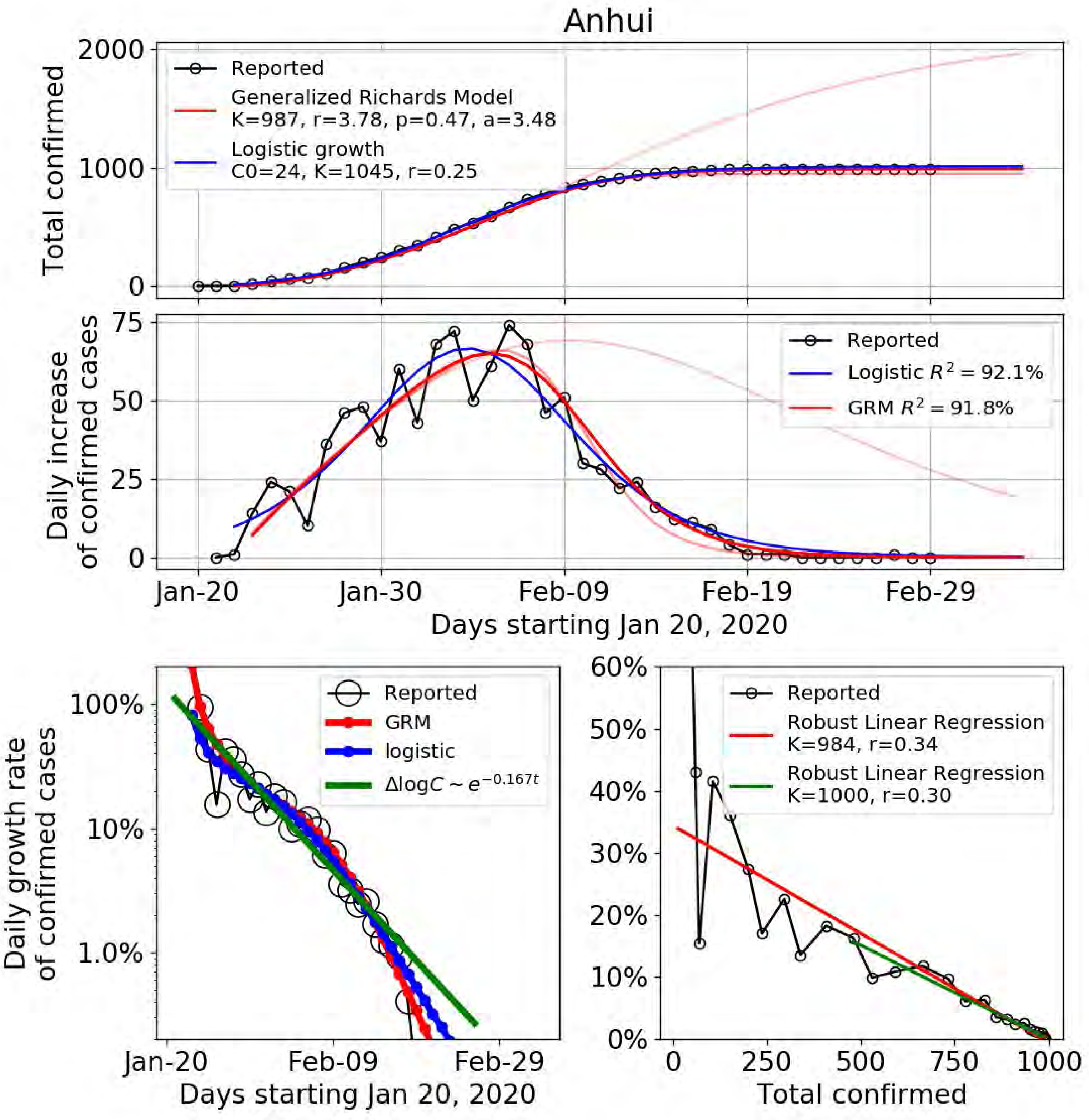

Anhui
Total confirmed
Reported
Generalized Richards Model
K=987, r=3.78, p=0.47, a=3.48
Logistic growth
C0=24, K=1045, r=0.25
Daily increase of confirmed cases
Logistic $R^2 = 92.1\%$
GRM $R^2 = 91.8\%$
Jan-20
Jan-30
Feb-09
Feb-19
Feb-29
Days starting Jan 20, 2020
Daily growth rate of confirmed cases
GRM
logistic
$\Delta \log C \sim e^{-0.167t}$
Robust Linear Regression
K=984, r=0.34
Robust Linear Regression
K=1000, r=0.30
Total confirmed

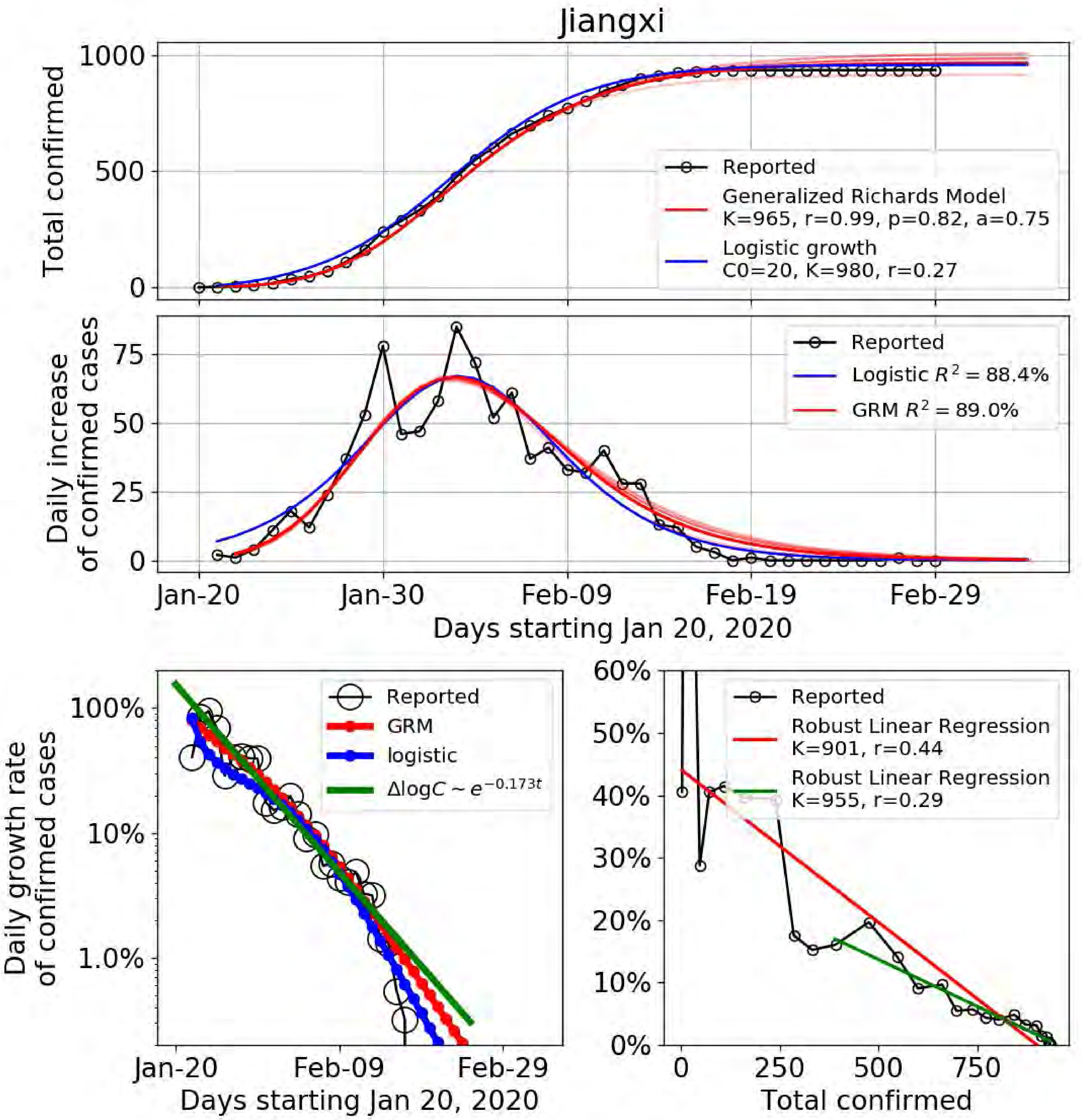

Jiangxi
Total confirmed
Reported
Generalized Richards Model
K=965, r=0.99, p=0.82, a=0.75
Logistic growth
C0=20, K=980, r=0.27
Daily increase of confirmed cases
Logistic $R^2 = 88.4\%$
GRM $R^2 = 89.0\%$
Jan-20
Jan-30
Feb-09
Feb-19
Feb-29
Days starting Jan 20, 2020
Daily growth rate of confirmed cases
GRM
logistic
$\Delta \log C \sim e^{-0.173t}$
Robust Linear Regression
K=901, r=0.44
Robust Linear Regression
K=955, r=0.29
Total confirmed

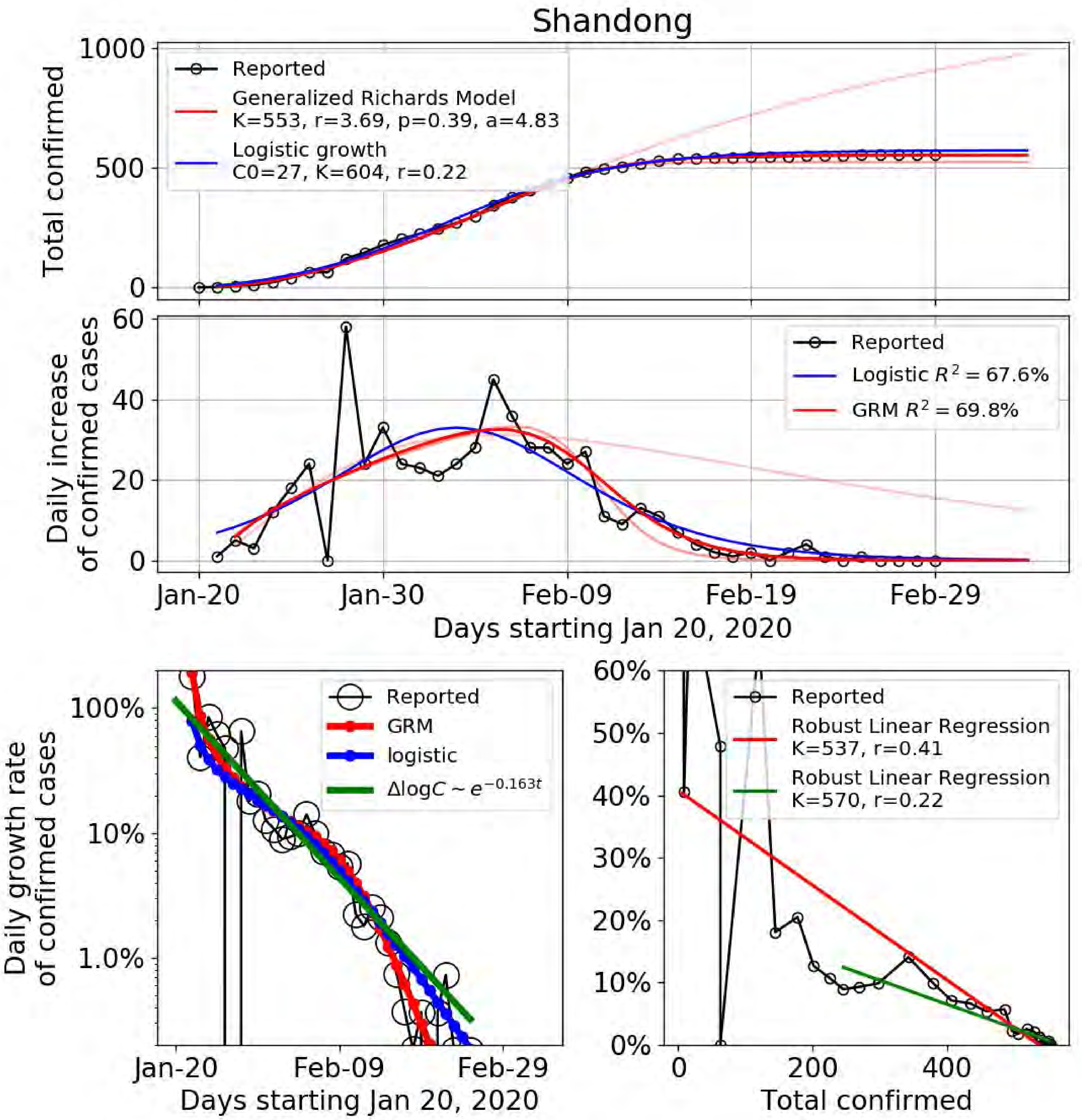
Shandong
Total confirmed
Reported
Generalized Richards Model
K=553, r=3.69, p=0.39, a=4.83
Logistic growth
C0=27, K=604, r=0.22
Daily increase of confirmed cases
Logistic $R^2 = 67.6\%$
GRM $R^2 = 69.8\%$
Jan-20
Jan-30
Feb-09
Feb-19
Feb-29
Days starting Jan 20, 2020
Daily growth rate of confirmed cases
GRM
logistic
$\Delta \log C \sim e^{-0.163t}$
Robust Linear Regression
K=537, r=0.41
Robust Linear Regression
K=570, r=0.22
Total confirmed

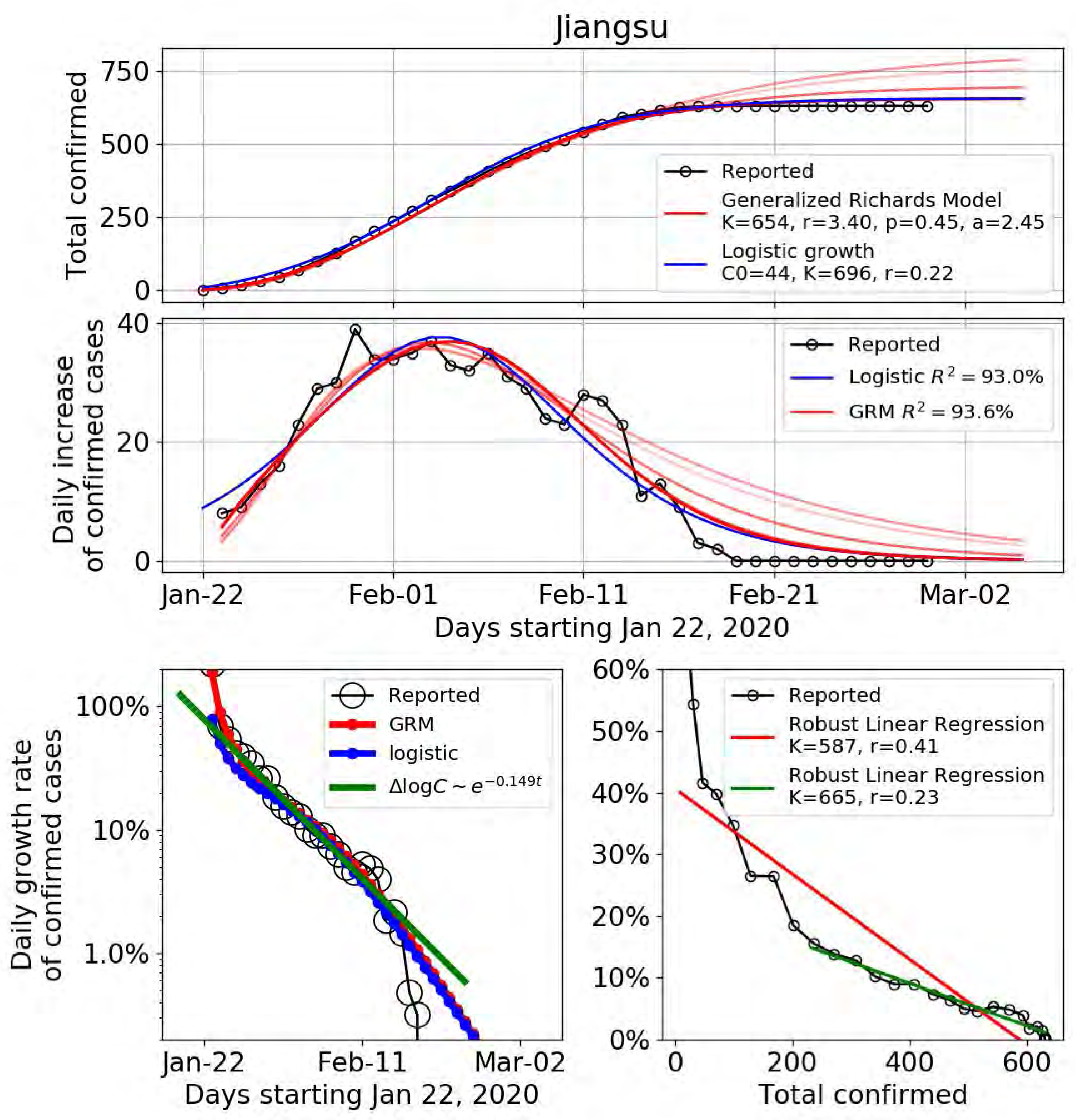

Jiangsu
Total confirmed
750
500
250
0
Reported
Generalized Richards Model
K=654, r=3.40, p=0.45, a=2.45
Logistic growth
C0=44, K=696, r=0.22
Daily increase of confirmed cases
40
20
0
Reported
Logistic $R^2 = 93.0\%$
GRM $R^2 = 93.6\%$
Jan-22
Feb-01
Feb-11
Feb-21
Mar-02
Days starting Jan 22, 2020
Daily growth rate of confirmed cases
100%
10%
1.0%
Reported
GRM
logistic
$\Delta \log C \sim e^{-0.149t}$
Jan-22
Feb-11
Mar-02
Days starting Jan 22, 2020
60%
50%
40%
30%
20%
10%
0%
Reported
Robust Linear Regression
K=587, r=0.41
Robust Linear Regression
K=665, r=0.23
0
200
400
600
Total confirmed

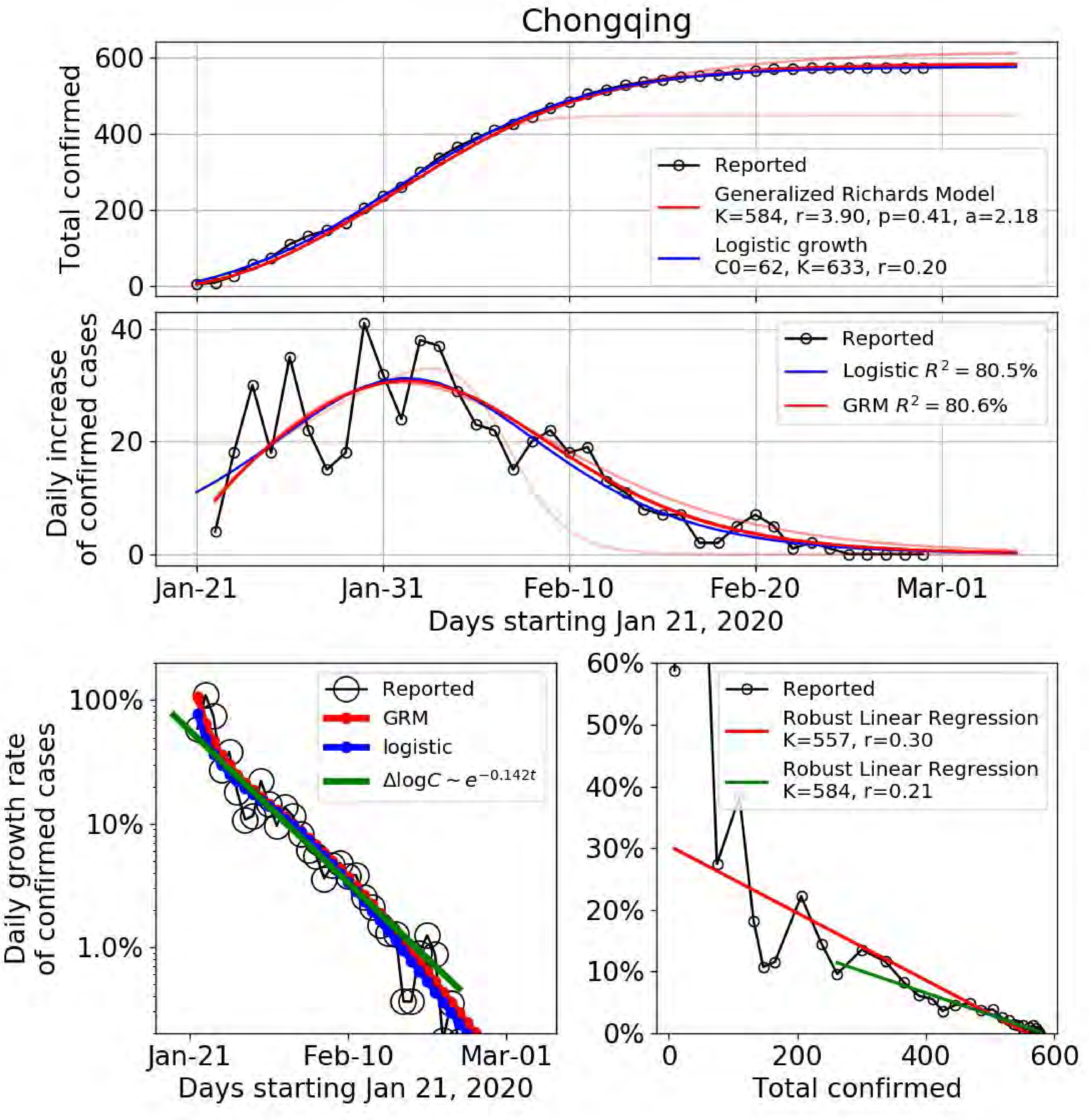

Chongqing
Total confirmed
Reported
Generalized Richards Model
K=584, r=3.90, p=0.41, a=2.18
Logistic growth
C0=62, K=633, r=0.20
Daily increase
of confirmed cases
Reported
Logistic $R^2 = 80.5\%$
GRM $R^2 = 80.6\%$
Jan-21
Jan-31
Feb-10
Feb-20
Mar-01
Days starting Jan 21, 2020
Daily growth rate
of confirmed cases
Reported
GRM
logistic
$\Delta \log C \sim e^{-0.142t}$
Days starting Jan 21, 2020
Reported
Robust Linear Regression
K=557, r=0.30
Robust Linear Regression
K=584, r=0.21
Total confirmed

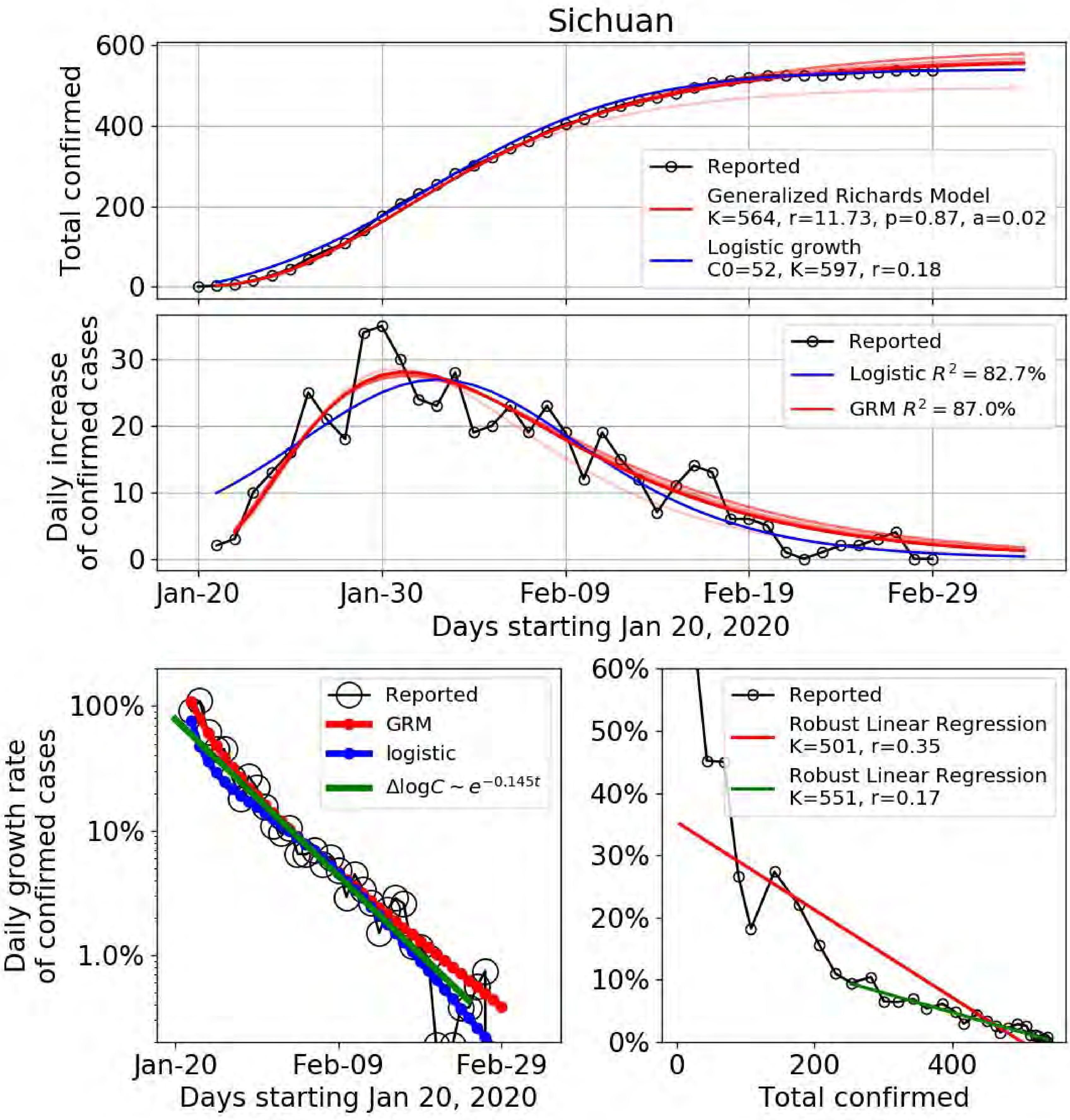
Sichuan
Total confirmed
Reported
Generalized Richards Model
K=564, r=11.73, p=0.87, a=0.02
Logistic growth
C0=52, K=597, r=0.18
Daily increase of confirmed cases
Logistic $R^2 = 82.7\%$
GRM $R^2 = 87.0\%$
Jan-20
Jan-30
Feb-09
Feb-19
Feb-29
Days starting Jan 20, 2020
Daily growth rate of confirmed cases
GRM
logistic
$\Delta \log C \sim e^{-0.145t}$
Robust Linear Regression
K=501, r=0.35
Robust Linear Regression
K=551, r=0.17
Total confirmed

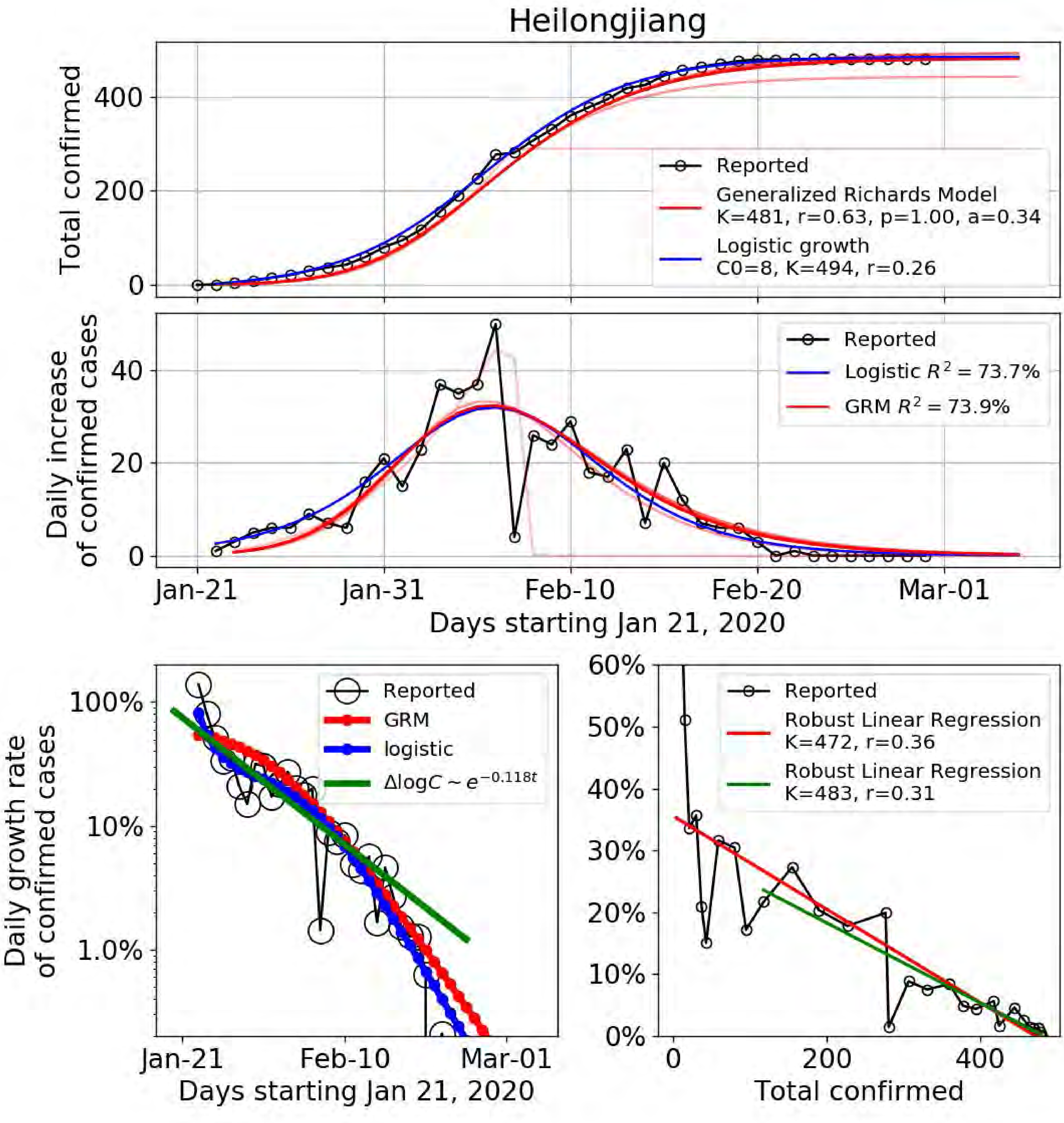

Heilongjiang
Total confirmed
0
200
400
Reported
Generalized Richards Model
K=481, r=0.63, p=1.00, a=0.34
Logistic growth
C0=8, K=494, r=0.26
Daily increase
of confirmed cases
0
20
40
Reported
Logistic $R^2 = 73.7\%$
GRM $R^2 = 73.9\%$
Jan-21
Jan-31
Feb-10
Feb-20
Mar-01
Days starting Jan 21, 2020
Daily growth rate
of confirmed cases
100%
10%
1.0%
Reported
GRM
logistic
$\Delta \log C \sim e^{-0.118t}$
Jan-21
Feb-10
Mar-01
Days starting Jan 21, 2020
60%
50%
40%
30%
20%
10%
0%
Reported
Robust Linear Regression
K=472, r=0.36
Robust Linear Regression
K=483, r=0.31
0
200
400
Total confirmed

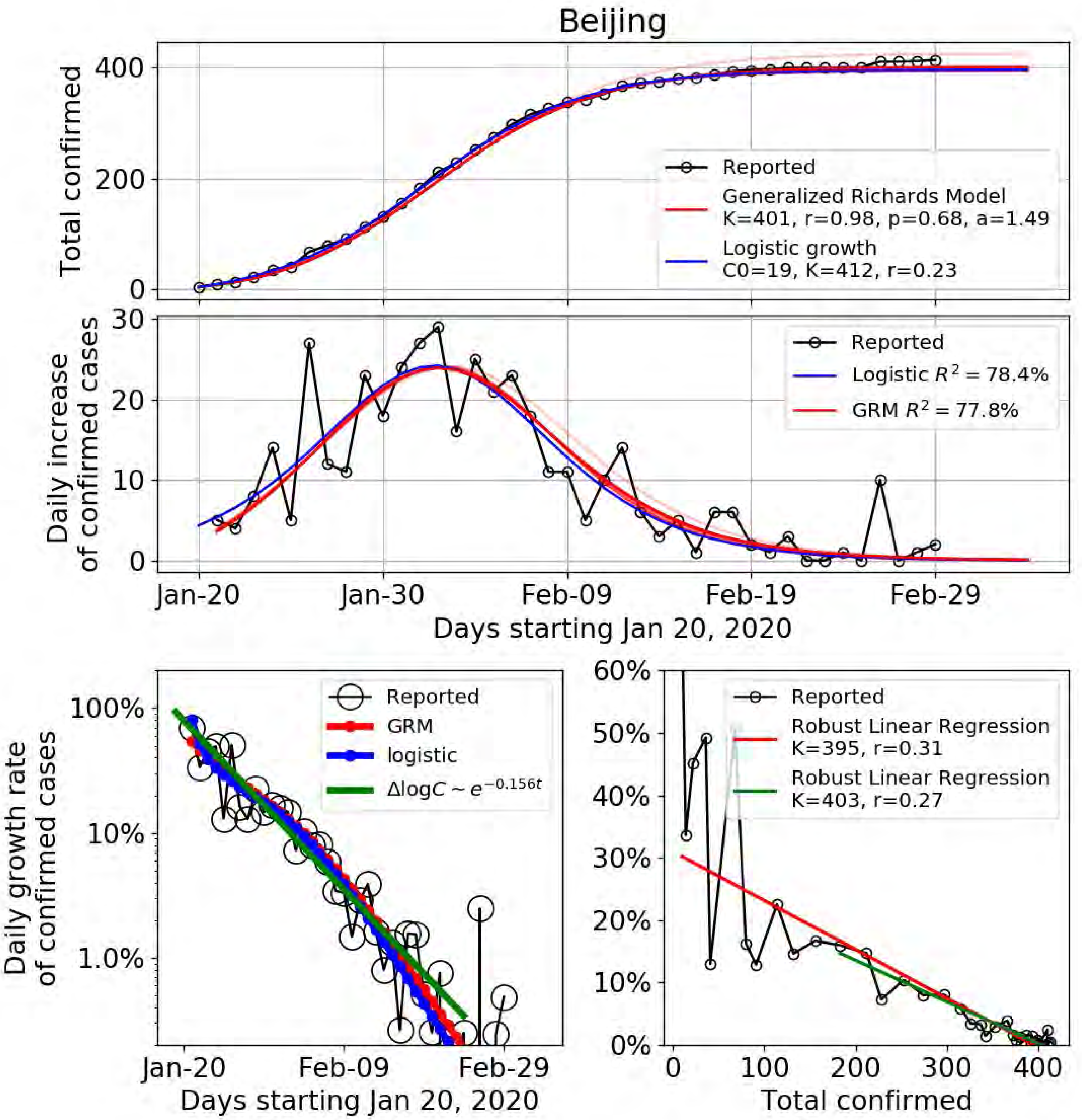

Beijing
Total confirmed
Reported
Generalized Richards Model
K=401, r=0.98, p=0.68, a=1.49
Logistic growth
C0=19, K=412, r=0.23
Daily increase of confirmed cases
Logistic $R^2 = 78.4\%$
GRM $R^2 = 77.8\%$
Jan-20
Jan-30
Feb-09
Feb-19
Feb-29
Days starting Jan 20, 2020
Daily growth rate of confirmed cases
GRM
logistic
$\Delta \log C \sim e^{-0.156t}$
Robust Linear Regression
K=395, r=0.31
Robust Linear Regression
K=403, r=0.27
Total confirmed

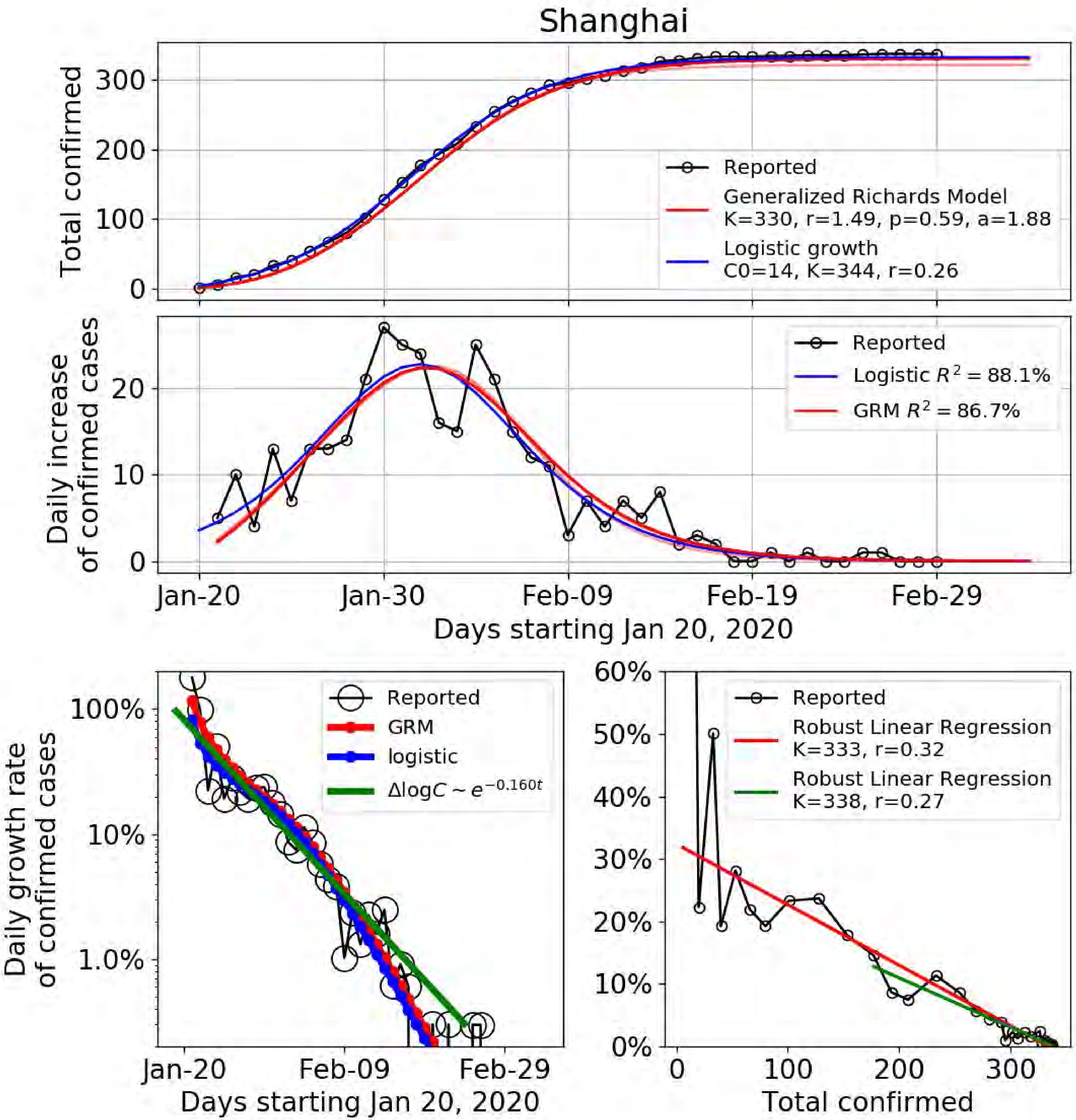

Shanghai
Total confirmed
Reported
Generalized Richards Model
K=330, r=1.49, p=0.59, a=1.88
Logistic growth
C0=14, K=344, r=0.26
Daily increase
of confirmed cases
Logistic $R^2 = 88.1\%$
GRM $R^2 = 86.7\%$
Jan-20
Jan-30
Feb-09
Feb-19
Feb-29
Days starting Jan 20, 2020
Daily growth rate
of confirmed cases
GRM
logistic
$\Delta \log C \sim e^{-0.160t}$
Robust Linear Regression
K=333, r=0.32
Robust Linear Regression
K=338, r=0.27
Total confirmed

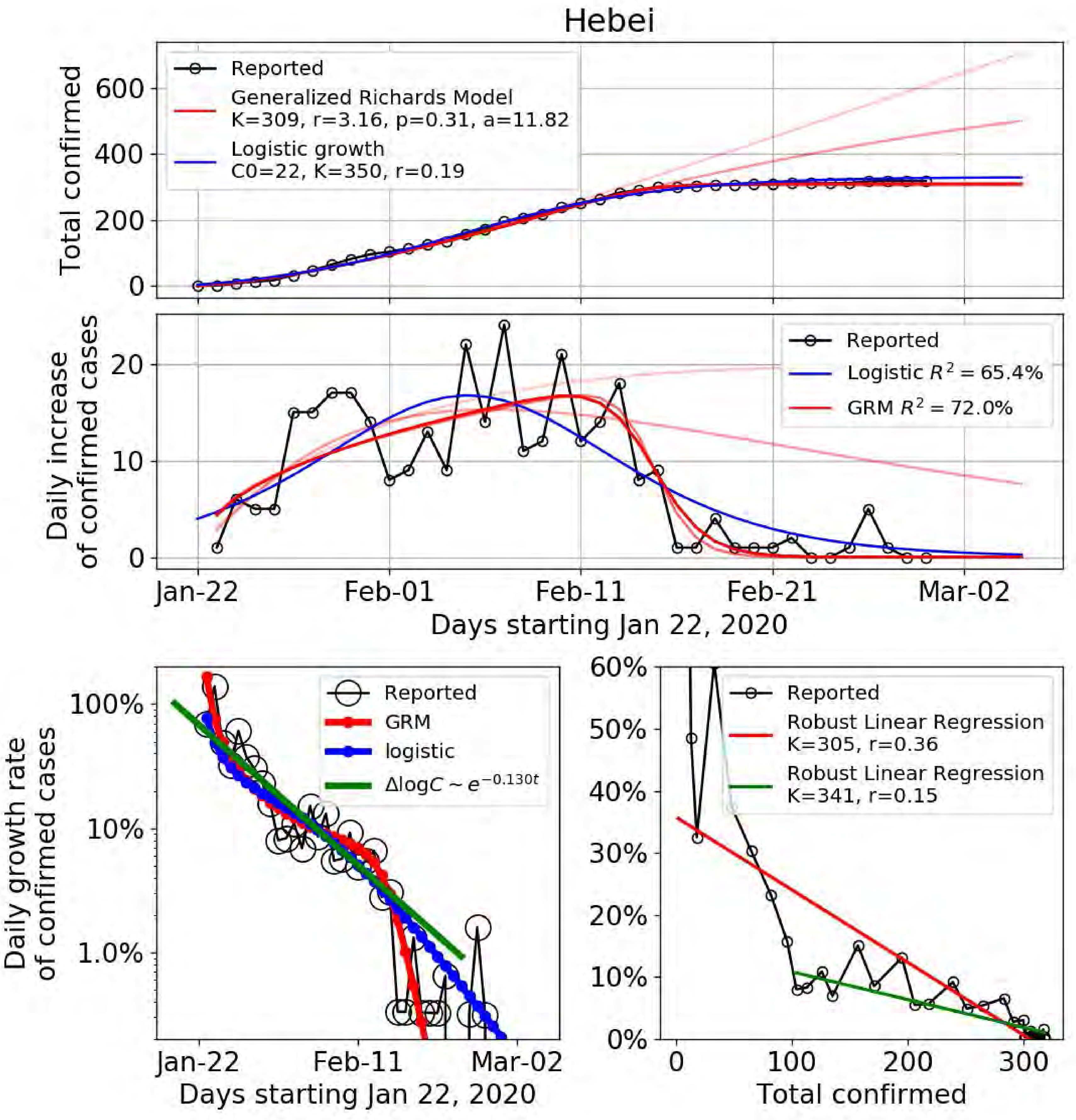
Hebei
Reported
Generalized Richards Model
K=309, r=3.16, p=0.31, a=11.82
Logistic growth
C0=22, K=350, r=0.19
Total confirmed
Daily increase of confirmed cases
Logistic $R^2 = 65.4\%$
GRM $R^2 = 72.0\%$
Jan-22
Feb-01
Feb-11
Feb-21
Mar-02
Days starting Jan 22, 2020
Daily growth rate of confirmed cases
GRM
logistic
$\Delta \log C \sim e^{-0.130t}$
Robust Linear Regression
K=305, r=0.36
Robust Linear Regression
K=341, r=0.15
Total confirmed

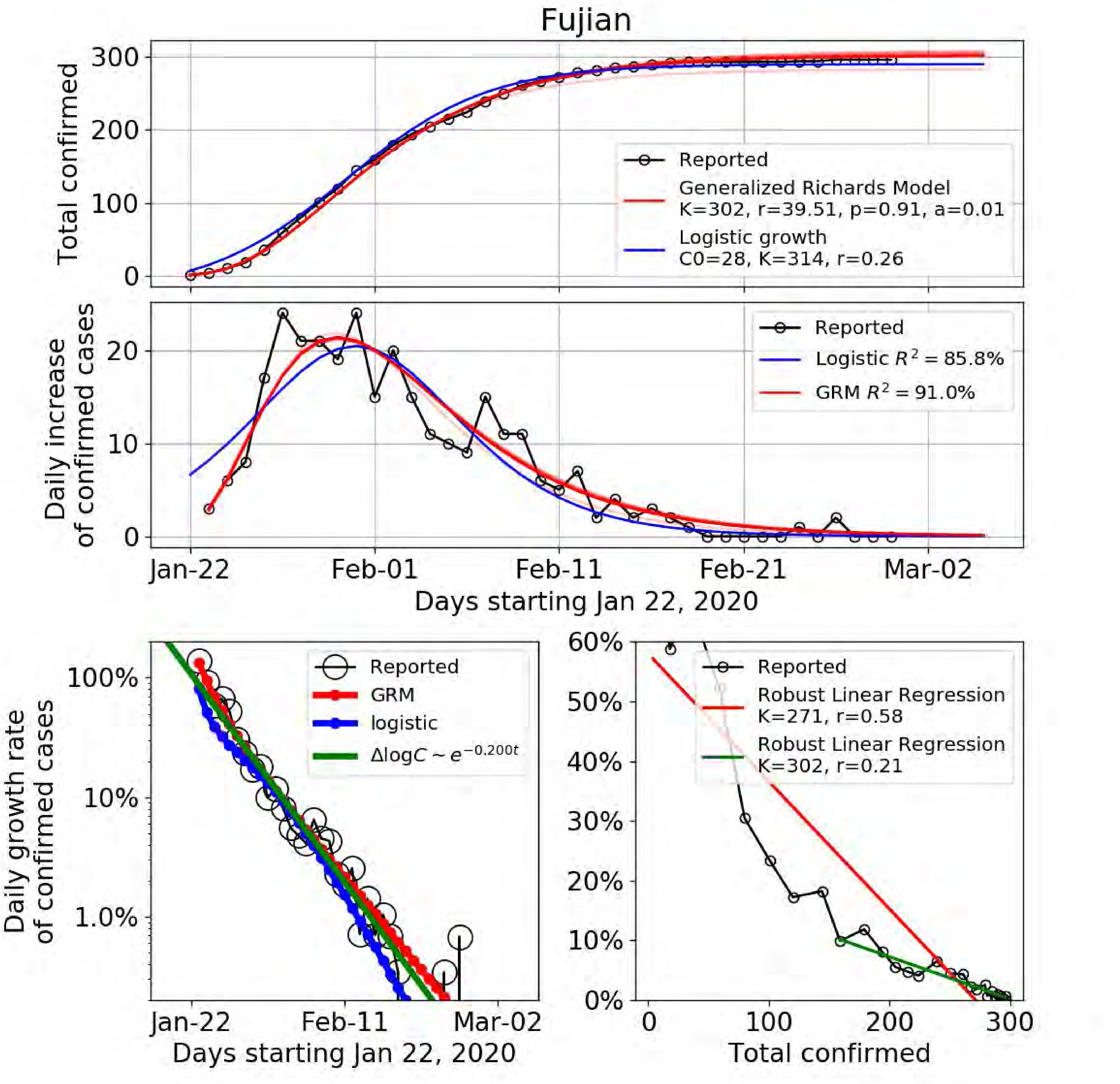

Fujian
Total confirmed
Reported
Generalized Richards Model
K=302, r=39.51, p=0.91, a=0.01
Logistic growth
C0=28, K=314, r=0.26
Daily increase of confirmed cases
Logistic $R^2 = 85.8\%$
GRM $R^2 = 91.0\%$
Jan-22
Feb-01
Feb-11
Feb-21
Mar-02
Days starting Jan 22, 2020
Daily growth rate of confirmed cases
GRM
logistic
$\Delta \log C \sim e^{-0.200t}$
Robust Linear Regression
K=271, r=0.58
Robust Linear Regression
K=302, r=0.21
Total confirmed

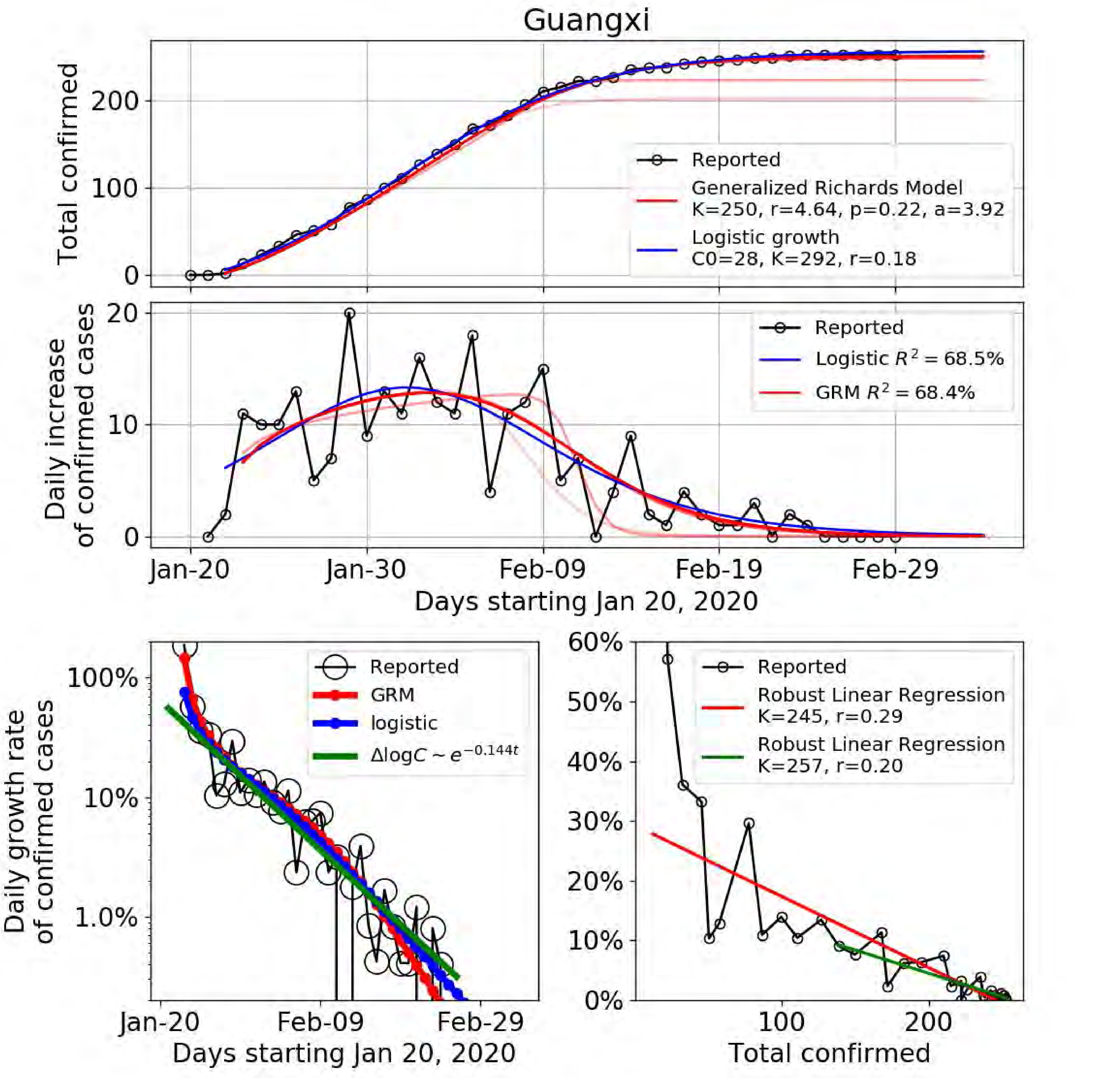

Guangxi
Total confirmed
Reported
Generalized Richards Model
K=250, r=4.64, p=0.22, a=3.92
Logistic growth
C0=28, K=292, r=0.18
Daily increase of confirmed cases
Reported
Logistic $R^2 = 68.5\%$
GRM $R^2 = 68.4\%$
Jan-20
Jan-30
Feb-09
Feb-19
Feb-29
Days starting Jan 20, 2020
Daily growth rate of confirmed cases
Reported
GRM
logistic
$\Delta \log C \sim e^{-0.144t}$
100%
10%
1.0%
Jan-20
Feb-09
Feb-29
Days starting Jan 20, 2020
60%
50%
40%
30%
20%
10%
0%
Reported
Robust Linear Regression
K=245, r=0.29
Robust Linear Regression
K=257, r=0.20
100
200
Total confirmed

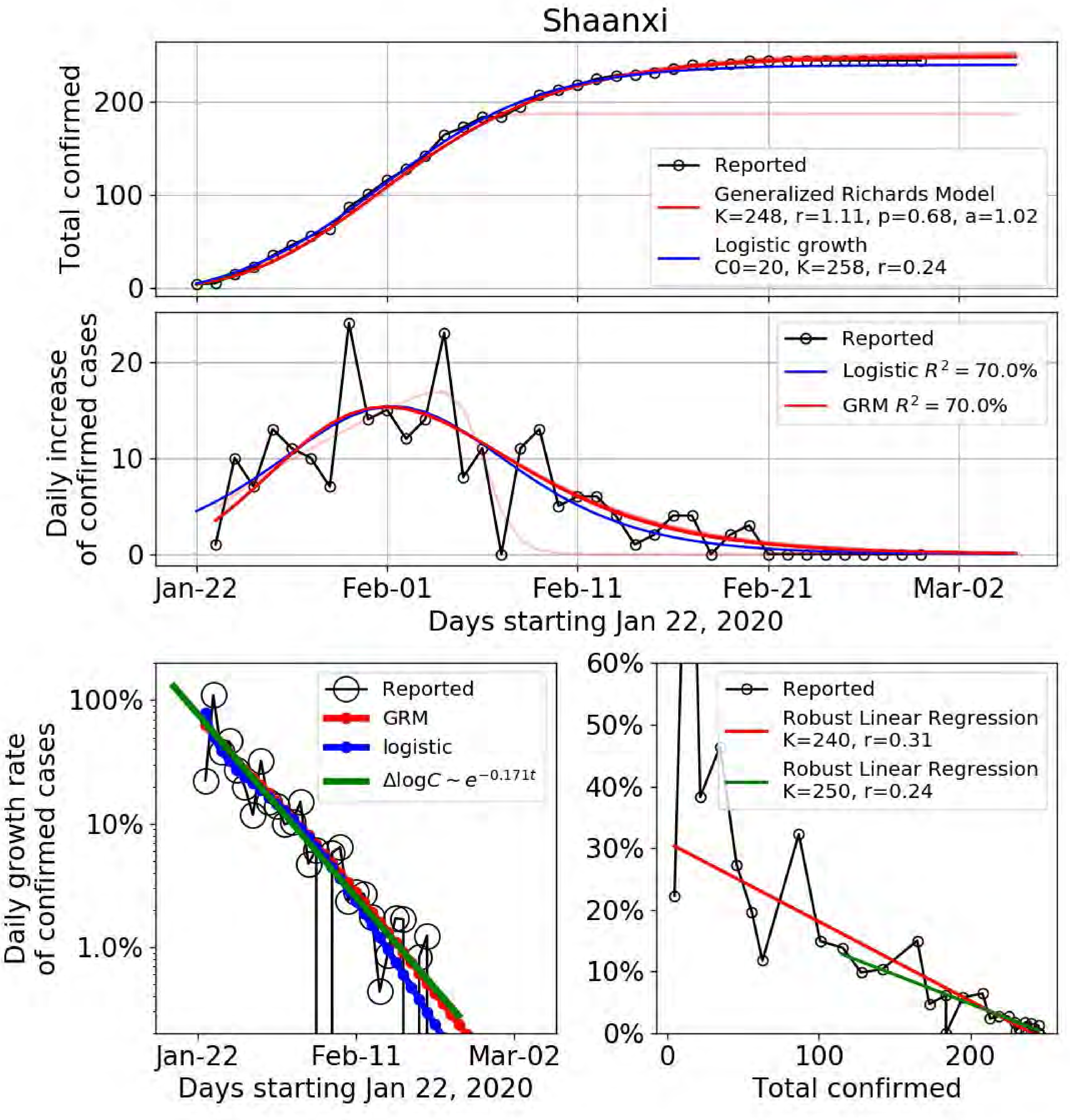
Shaanxi
Total confirmed
Reported
Generalized Richards Model
K=248, r=1.11, p=0.68, a=1.02
Logistic growth
C0=20, K=258, r=0.24
Daily increase of confirmed cases
Reported
Logistic $R^2 = 70.0\%$
GRM $R^2 = 70.0\%$
Jan-22
Feb-01
Feb-11
Feb-21
Mar-02
Days starting Jan 22, 2020
Daily growth rate of confirmed cases
Reported
GRM
logistic
$\Delta \log C \sim e^{-0.171t}$
Jan-22
Feb-11
Mar-02
Days starting Jan 22, 2020
Reported
Robust Linear Regression
K=240, r=0.31
Robust Linear Regression
K=250, r=0.24
Total confirmed

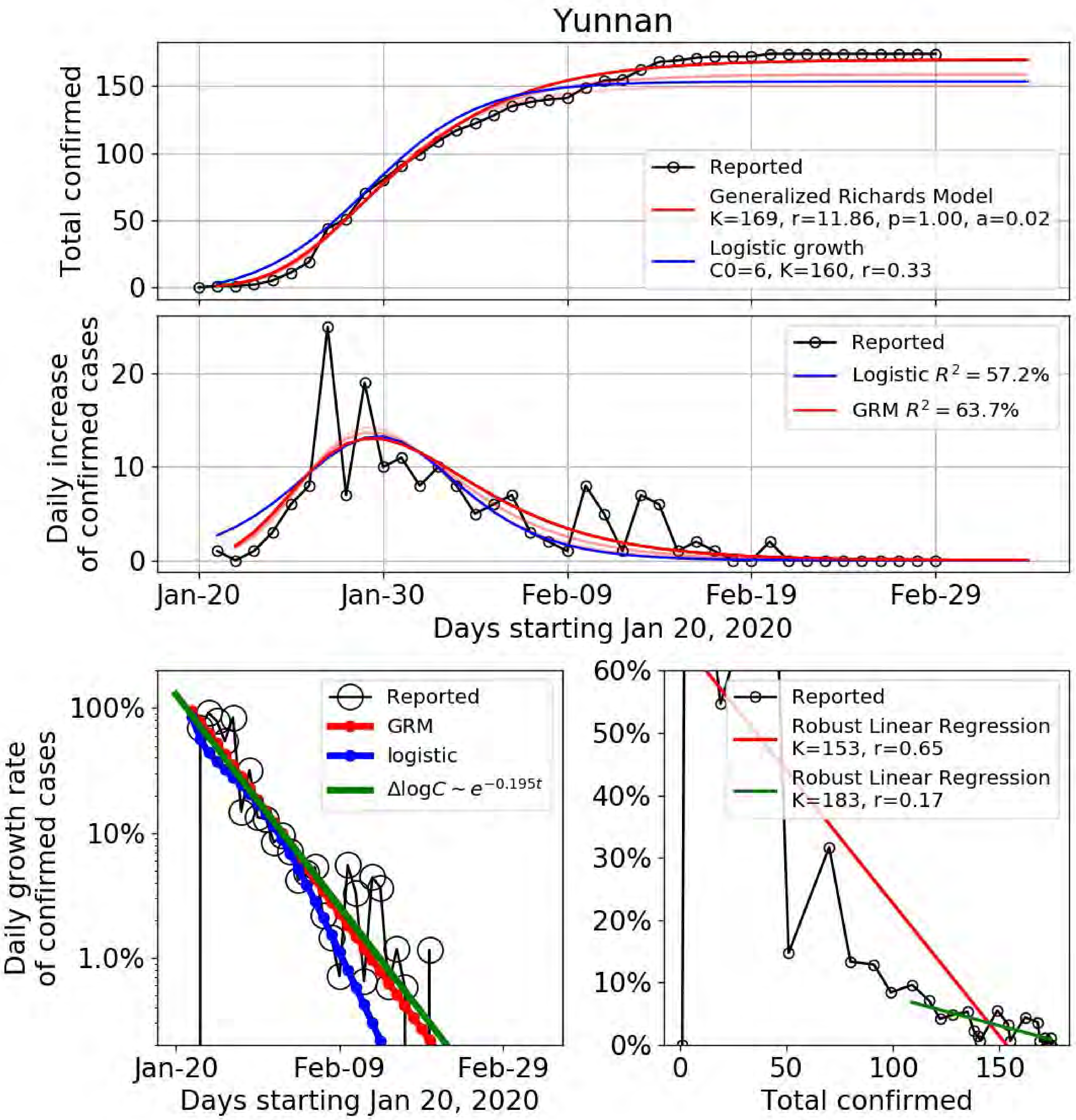
Yunnan
Total confirmed
Reported
Generalized Richards Model
K=169, r=11.86, p=1.00, a=0.02
Logistic growth
C0=6, K=160, r=0.33
Daily increase of confirmed cases
Logistic $R^2 = 57.2\%$
GRM $R^2 = 63.7\%$
Jan-20
Jan-30
Feb-09
Feb-19
Feb-29
Days starting Jan 20, 2020
Daily growth rate of confirmed cases
GRM
logistic
$\Delta \log C \sim e^{-0.195t}$
Robust Linear Regression
K=153, r=0.65
Robust Linear Regression
K=183, r=0.17
Total confirmed

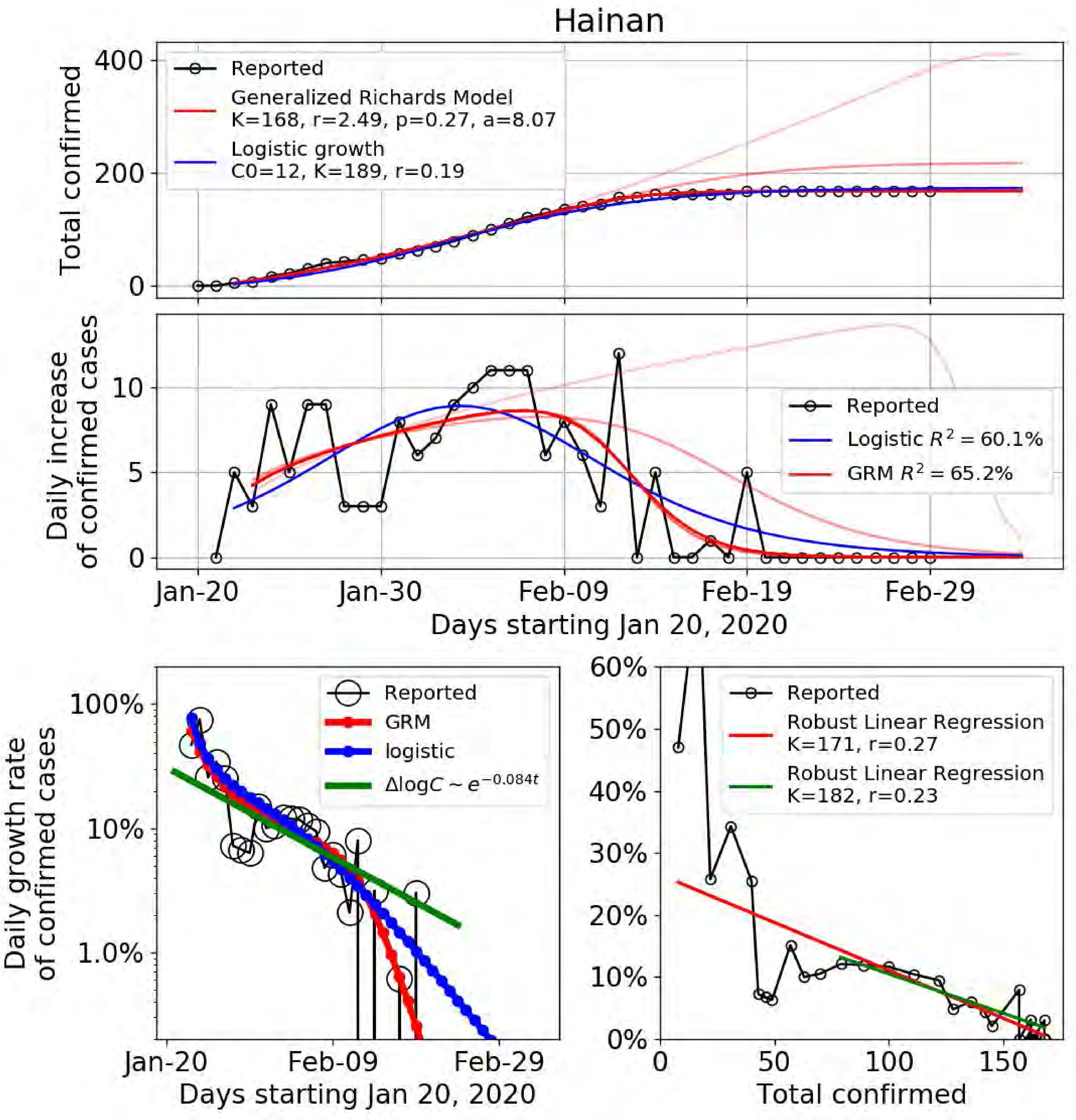

Hainan
Total confirmed
Reported
Generalized Richards Model
K=168, r=2.49, p=0.27, a=8.07
Logistic growth
C0=12, K=189, r=0.19
Daily increase of confirmed cases
Reported
Logistic $R^2 = 60.1\%$
GRM $R^2 = 65.2\%$
Jan-20
Jan-30
Feb-09
Feb-19
Feb-29
Days starting Jan 20, 2020
Daily growth rate of confirmed cases
Reported
GRM
logistic
$\Delta \log C \sim e^{-0.084t}$
Reported
Robust Linear Regression
K=171, r=0.27
Robust Linear Regression
K=182, r=0.23
Total confirmed

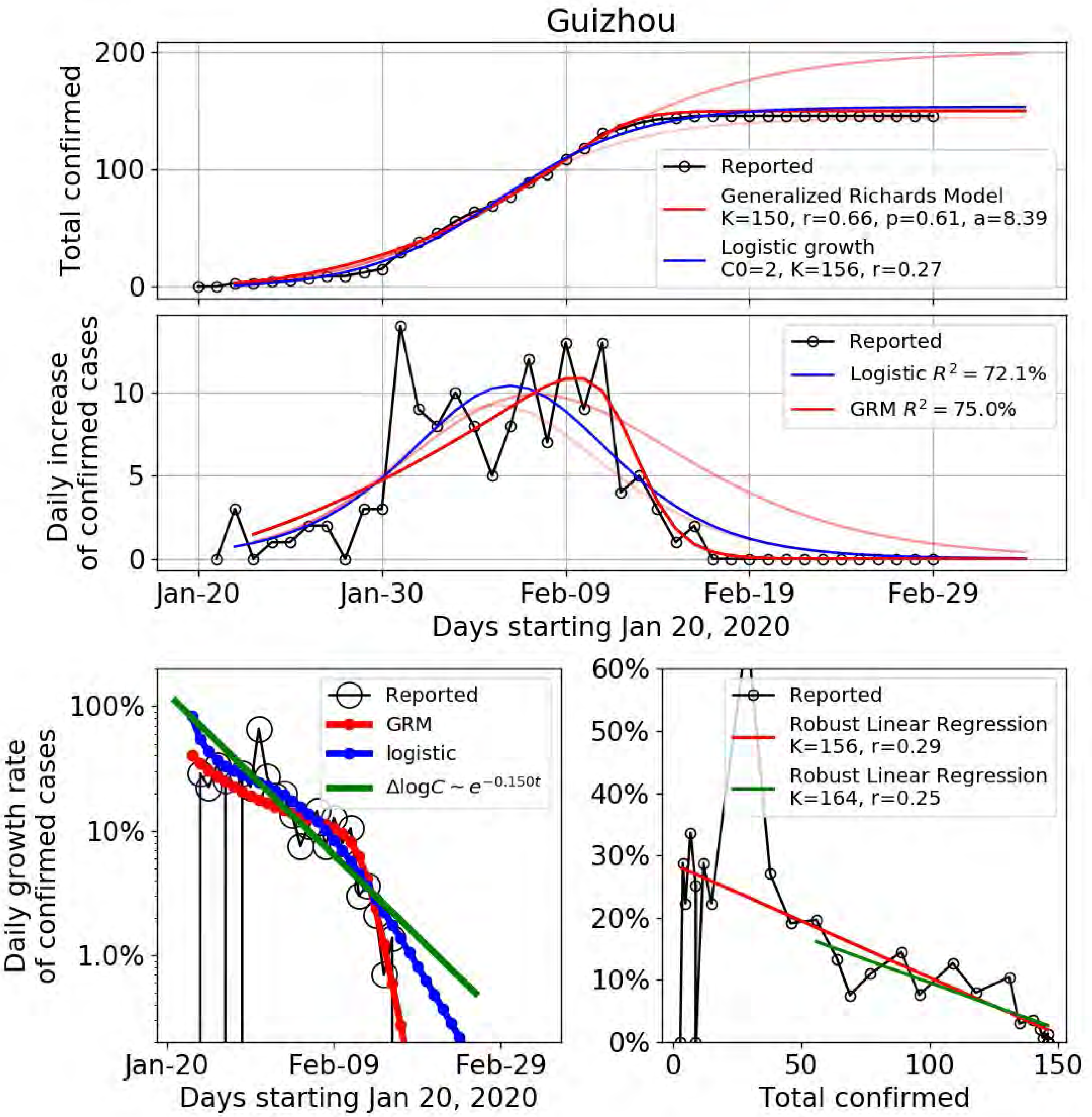

Guizhou
Total confirmed
200
100
0
Reported
Generalized Richards Model
K=150, r=0.66, p=0.61, a=8.39
Logistic growth
C0=2, K=156, r=0.27
Daily increase of confirmed cases
10
5
0
Reported
Logistic $R^2 = 72.1\%$
GRM $R^2 = 75.0\%$
Jan-20
Jan-30
Feb-09
Feb-19
Feb-29
Days starting Jan 20, 2020
Daily growth rate of confirmed cases
100%
10%
1.0%
Reported
GRM
logistic
$\Delta \log C \sim e^{-0.150t}$
Jan-20
Feb-09
Feb-29
Days starting Jan 20, 2020
60%
50%
40%
30%
20%
10%
0%
Reported
Robust Linear Regression
K=156, r=0.29
Robust Linear Regression
K=164, r=0.25
0
50
100
150
Total confirmed

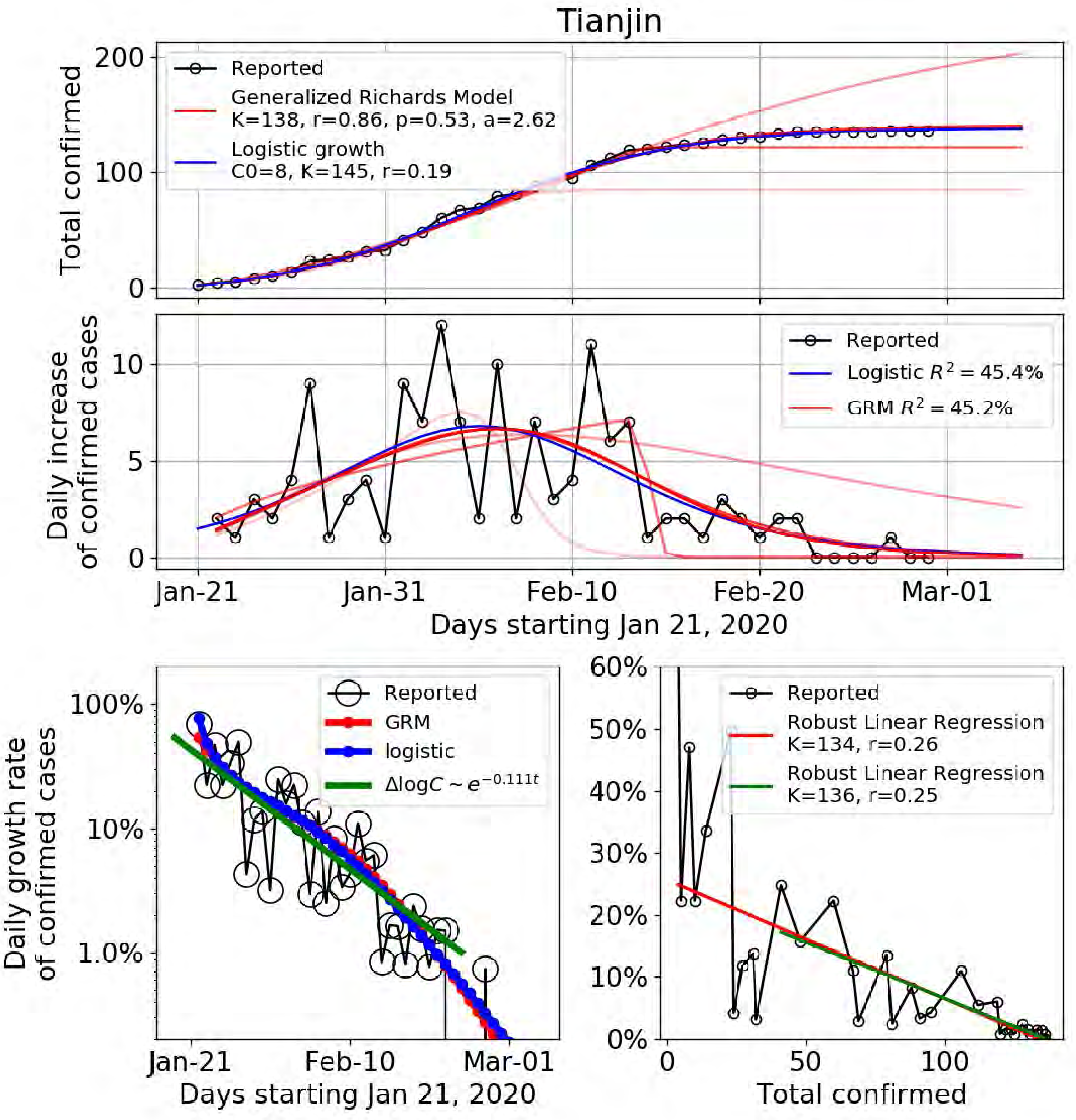

Tianjin
Total confirmed
Reported
Generalized Richards Model
K=138, r=0.86, p=0.53, a=2.62
Logistic growth
C0=8, K=145, r=0.19
Daily increase of confirmed cases
Logistic $R^2 = 45.4\%$
GRM $R^2 = 45.2\%$
Jan-21
Jan-31
Feb-10
Feb-20
Mar-01
Days starting Jan 21, 2020
Daily growth rate of confirmed cases
GRM
logistic
$\Delta \log C \sim e^{-0.111t}$
Robust Linear Regression
K=134, r=0.26
Robust Linear Regression
K=136, r=0.25
Total confirmed

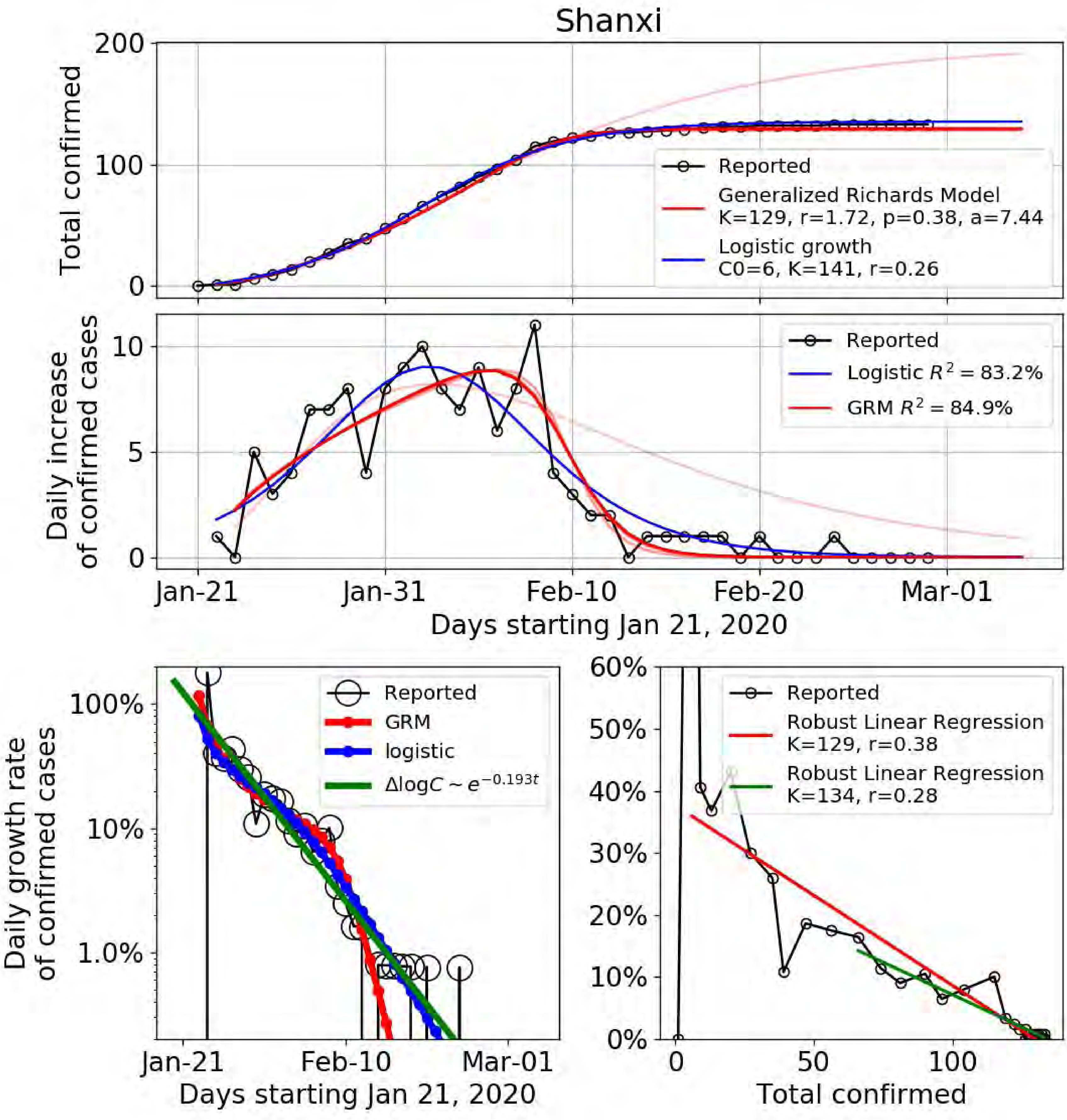

Shanxi
Total confirmed
200
100
0
Reported
Generalized Richards Model
K=129, r=1.72, p=0.38, a=7.44
Logistic growth
C0=6, K=141, r=0.26
Daily increase of confirmed cases
10
5
0
Reported
Logistic $R^2 = 83.2\%$
GRM $R^2 = 84.9\%$
Jan-21
Jan-31
Feb-10
Feb-20
Mar-01
Days starting Jan 21, 2020
Daily growth rate of confirmed cases
100%
10%
1.0%
Reported
GRM
logistic
$\Delta \log C \sim e^{-0.193t}$
Jan-21
Feb-10
Mar-01
Days starting Jan 21, 2020
60%
50%
40%
30%
20%
10%
0%
Reported
Robust Linear Regression
K=129, r=0.38
Robust Linear Regression
K=134, r=0.28
0
50
100
Total confirmed

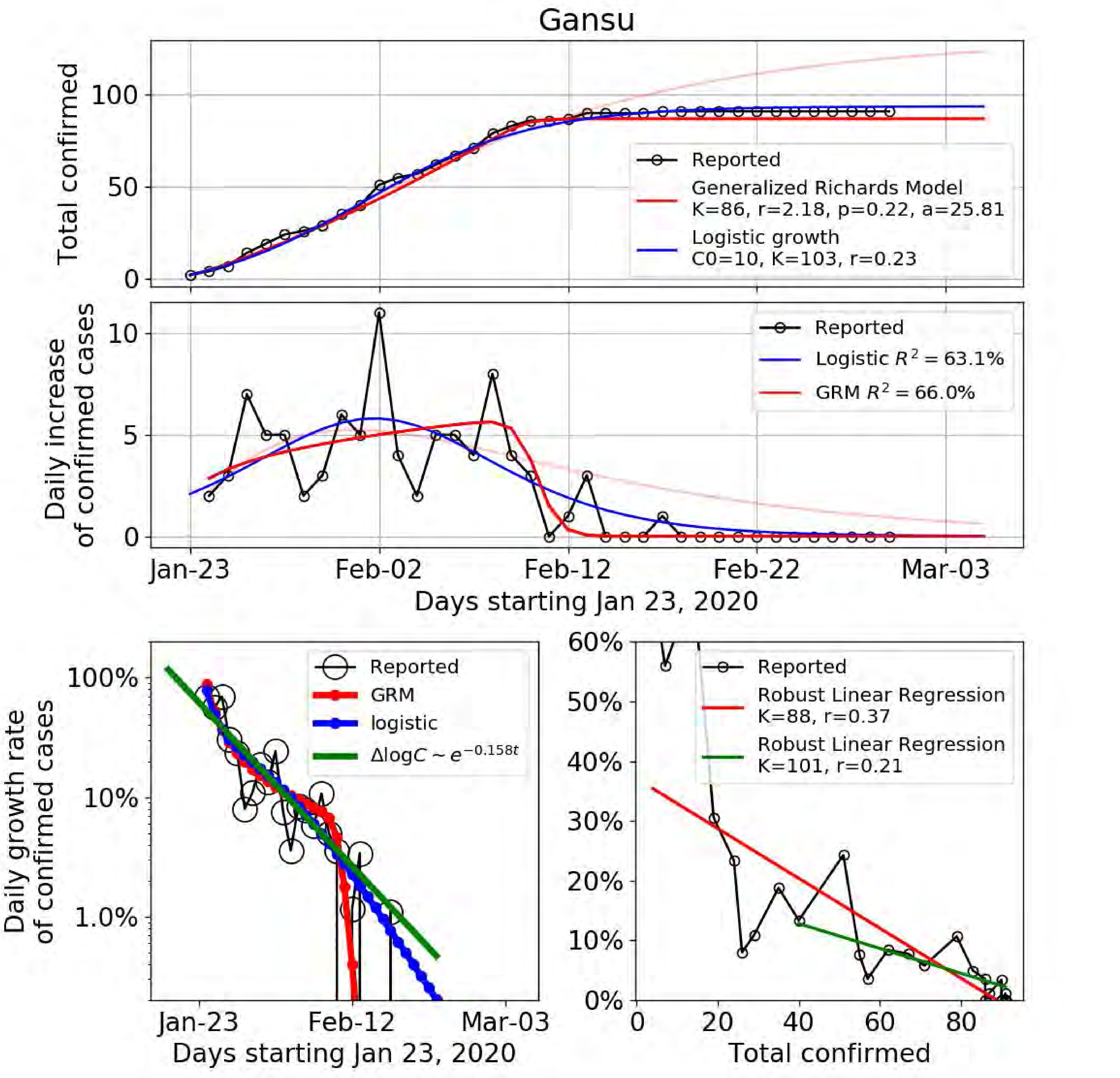

Gansu
Total confirmed
Reported
Generalized Richards Model
K=86, r=2.18, p=0.22, a=25.81
Logistic growth
C0=10, K=103, r=0.23
Daily increase of confirmed cases
Reported
Logistic $R^2 = 63.1\%$
GRM $R^2 = 66.0\%$
Jan-23
Feb-02
Feb-12
Feb-22
Mar-03
Days starting Jan 23, 2020
Daily growth rate of confirmed cases
Reported
GRM
logistic
$\Delta \log C \sim e^{-0.158t}$
Days starting Jan 23, 2020
Reported
Robust Linear Regression
K=88, r=0.37
Robust Linear Regression
K=101, r=0.21
Total confirmed

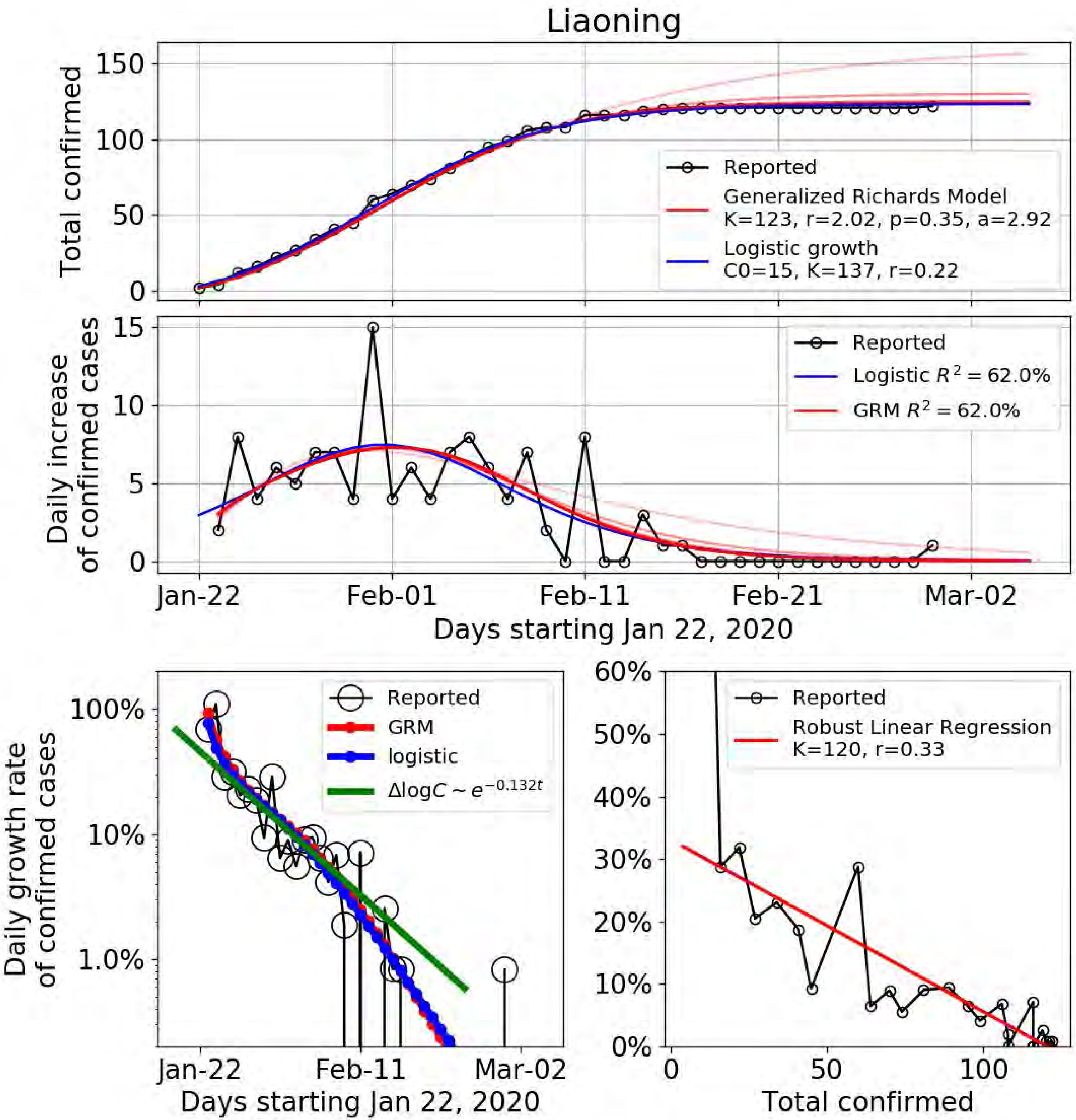

Liaoning
Total confirmed
Reported
Generalized Richards Model
K=123, r=2.02, p=0.35, a=2.92
Logistic growth
C0=15, K=137, r=0.22
Daily increase of confirmed cases
Logistic $R^2 = 62.0\%$
GRM $R^2 = 62.0\%$
Jan-22
Feb-01
Feb-11
Feb-21
Mar-02
Days starting Jan 22, 2020
Daily growth rate of confirmed cases
GRM
logistic
$\Delta \log C \sim e^{-0.132t}$
Robust Linear Regression
K=120, r=0.33
Total confirmed

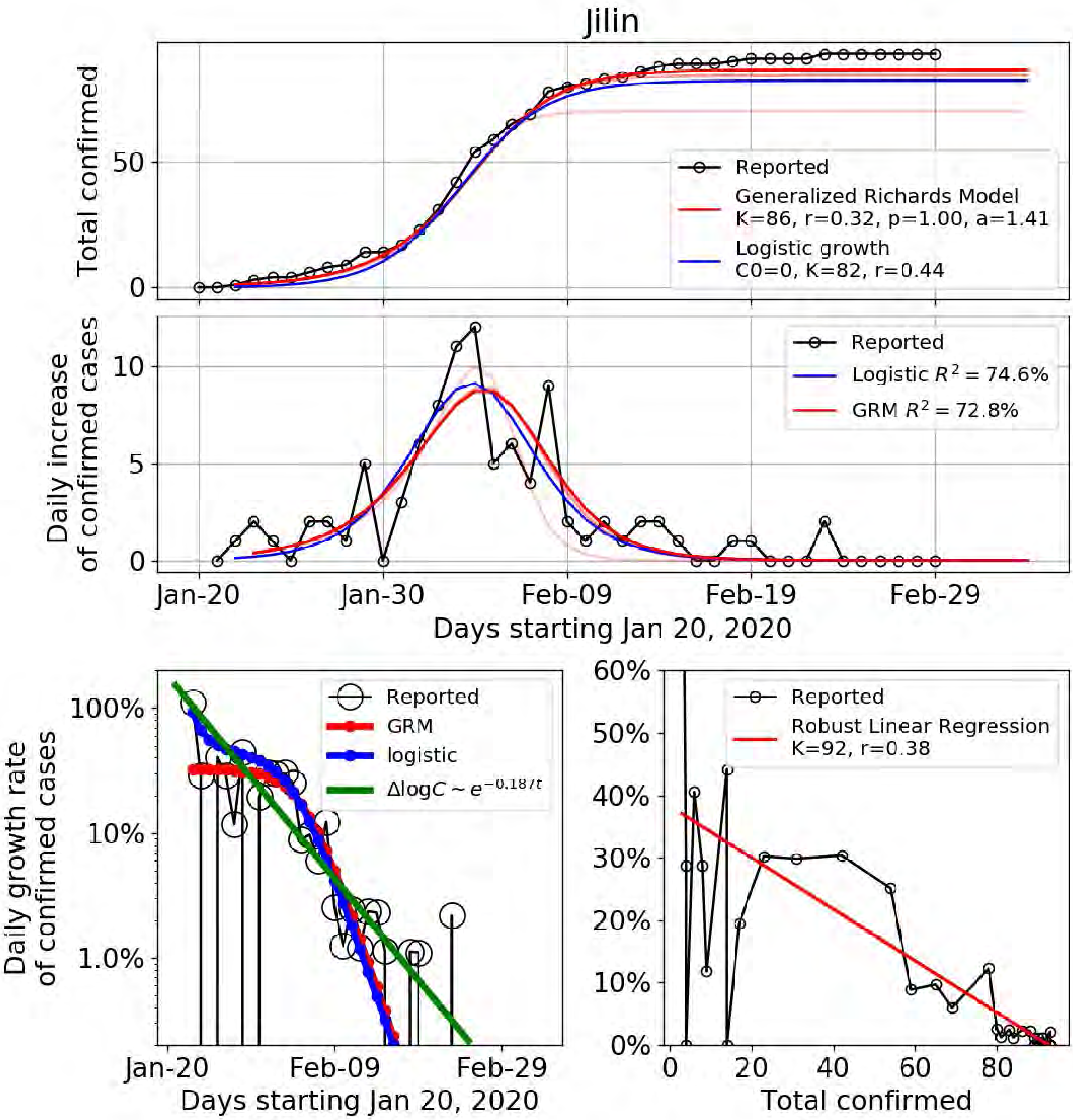
Jilin
Total confirmed
Reported
Generalized Richards Model
K=86, r=0.32, p=1.00, a=1.41
Logistic growth
C0=0, K=82, r=0.44
Daily increase of confirmed cases
Logistic $R^2 = 74.6\%$
GRM $R^2 = 72.8\%$
Jan-20
Jan-30
Feb-09
Feb-19
Feb-29
Days starting Jan 20, 2020
Daily growth rate of confirmed cases
GRM
logistic
$\Delta \log C \sim e^{-0.187t}$
Robust Linear Regression
K=92, r=0.38
Total confirmed

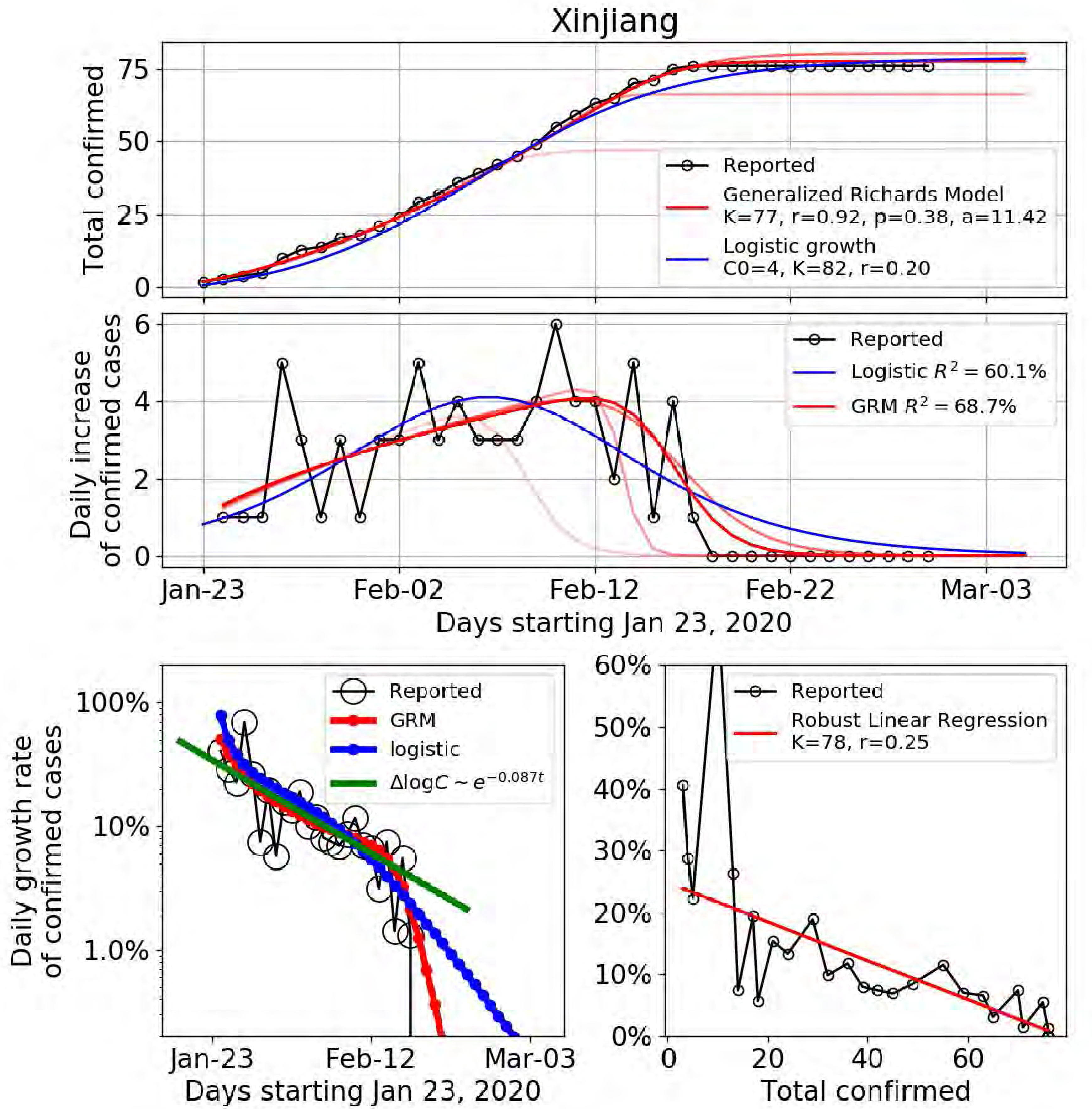

Xinjiang
Total confirmed
Reported
Generalized Richards Model
K=77, r=0.92, p=0.38, a=11.42
Logistic growth
C0=4, K=82, r=0.20
Daily increase of confirmed cases
Logistic $R^2 = 60.1\%$
GRM $R^2 = 68.7\%$
Jan-23
Feb-02
Feb-12
Feb-22
Mar-03
Days starting Jan 23, 2020
Daily growth rate of confirmed cases
GRM
logistic
$\Delta \log C \sim e^{-0.087t}$
Robust Linear Regression
K=78, r=0.25
Total confirmed

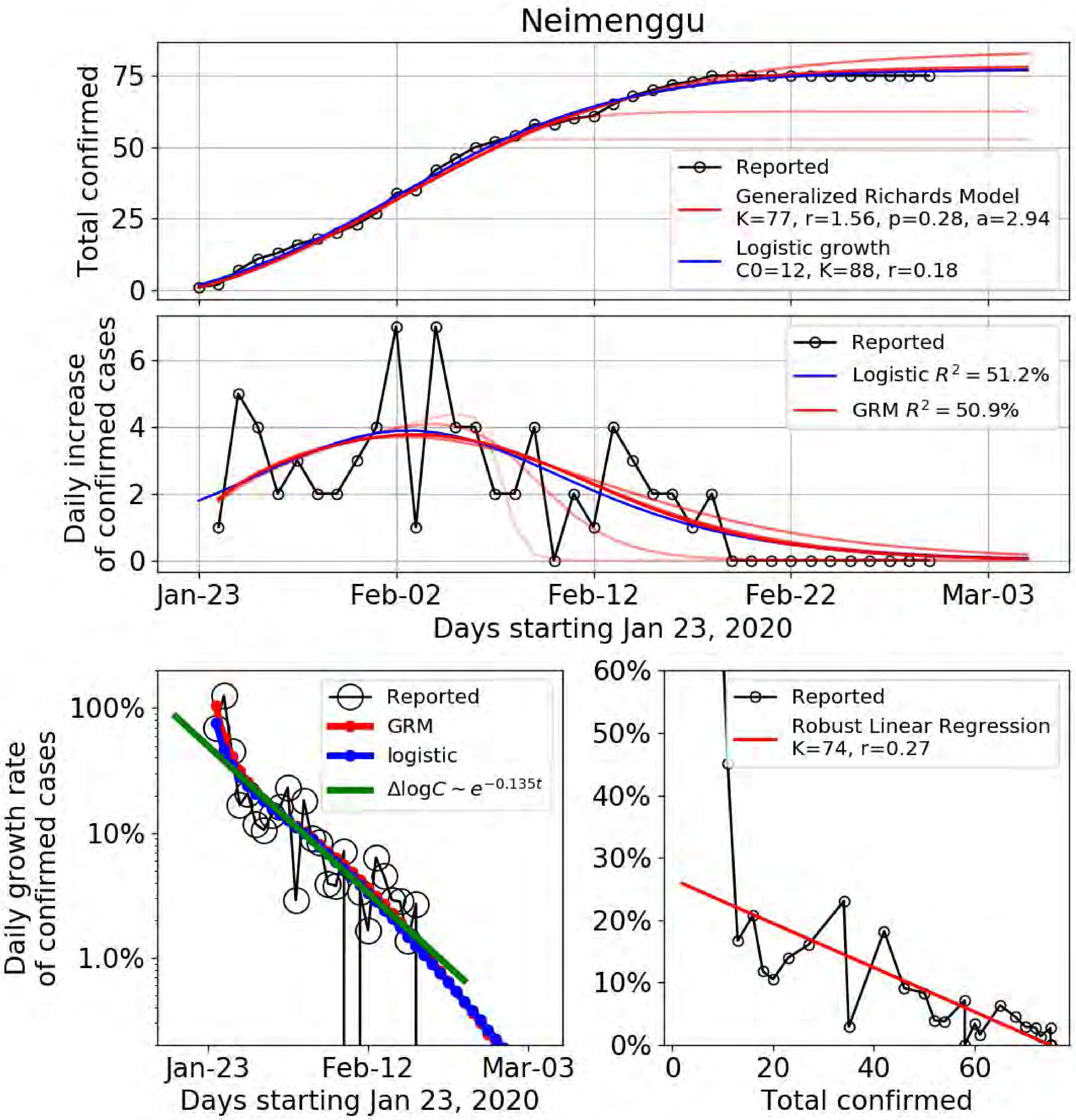

Neimenggu
Total confirmed
Reported
Generalized Richards Model
K=77, r=1.56, p=0.28, a=2.94
Logistic growth
C0=12, K=88, r=0.18
Daily increase of confirmed cases
Logistic $R^2 = 51.2\%$
GRM $R^2 = 50.9\%$
Jan-23
Feb-02
Feb-12
Feb-22
Mar-03
Days starting Jan 23, 2020
Daily growth rate of confirmed cases
GRM
logistic
$\Delta \log C \sim e^{-0.135t}$
Robust Linear Regression
K=74, r=0.27
Total confirmed

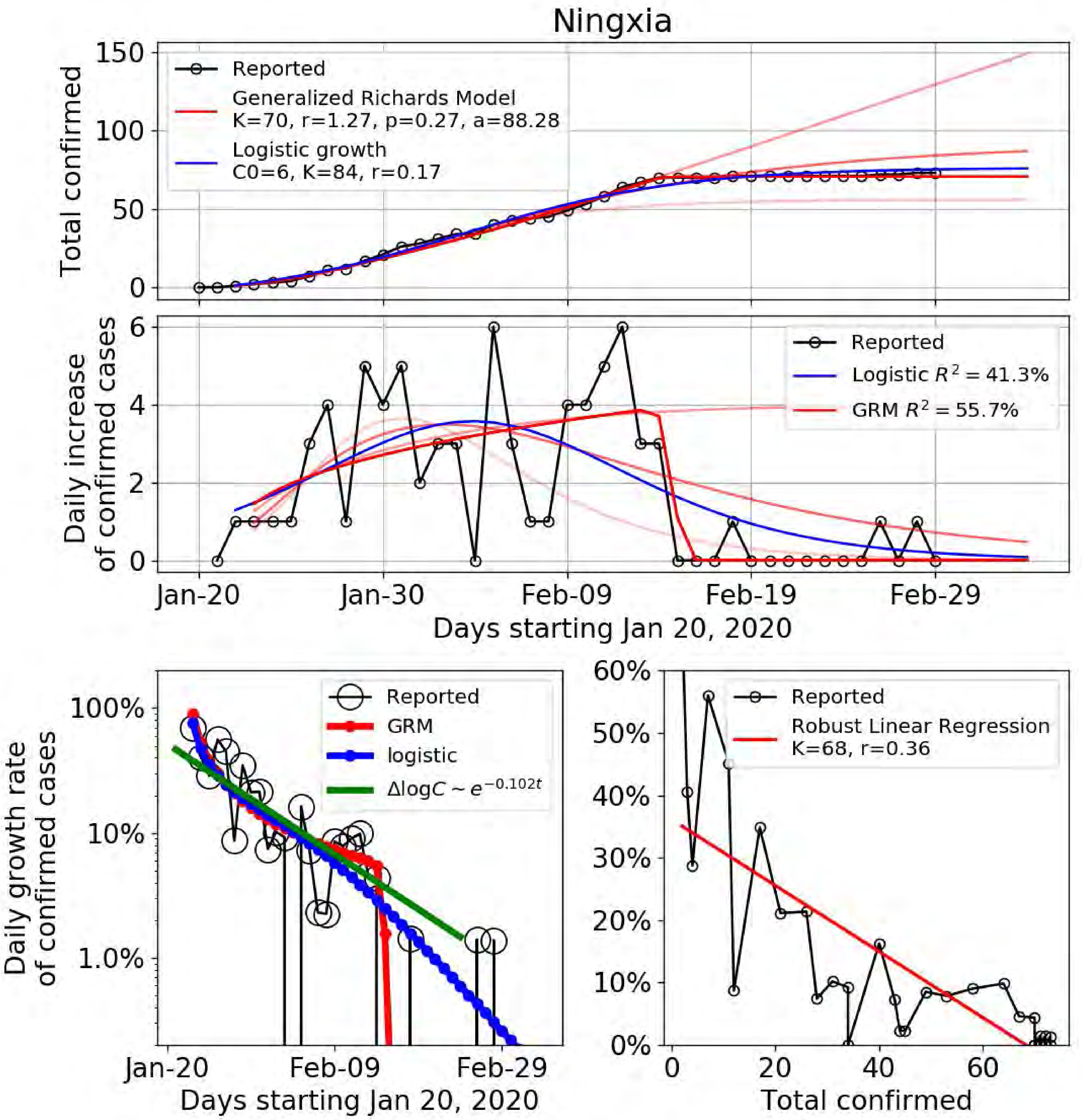

Ningxia
Total confirmed
Reported
Generalized Richards Model
K=70, r=1.27, p=0.27, a=88.28
Logistic growth
C0=6, K=84, r=0.17
Daily increase of confirmed cases
Logistic $R^2 = 41.3\%$
GRM $R^2 = 55.7\%$
Jan-20
Jan-30
Feb-09
Feb-19
Feb-29
Days starting Jan 20, 2020
Daily growth rate of confirmed cases
GRM
logistic
$\Delta \log C \sim e^{-0.102t}$
Robust Linear Regression
K=68, r=0.36
Total confirmed

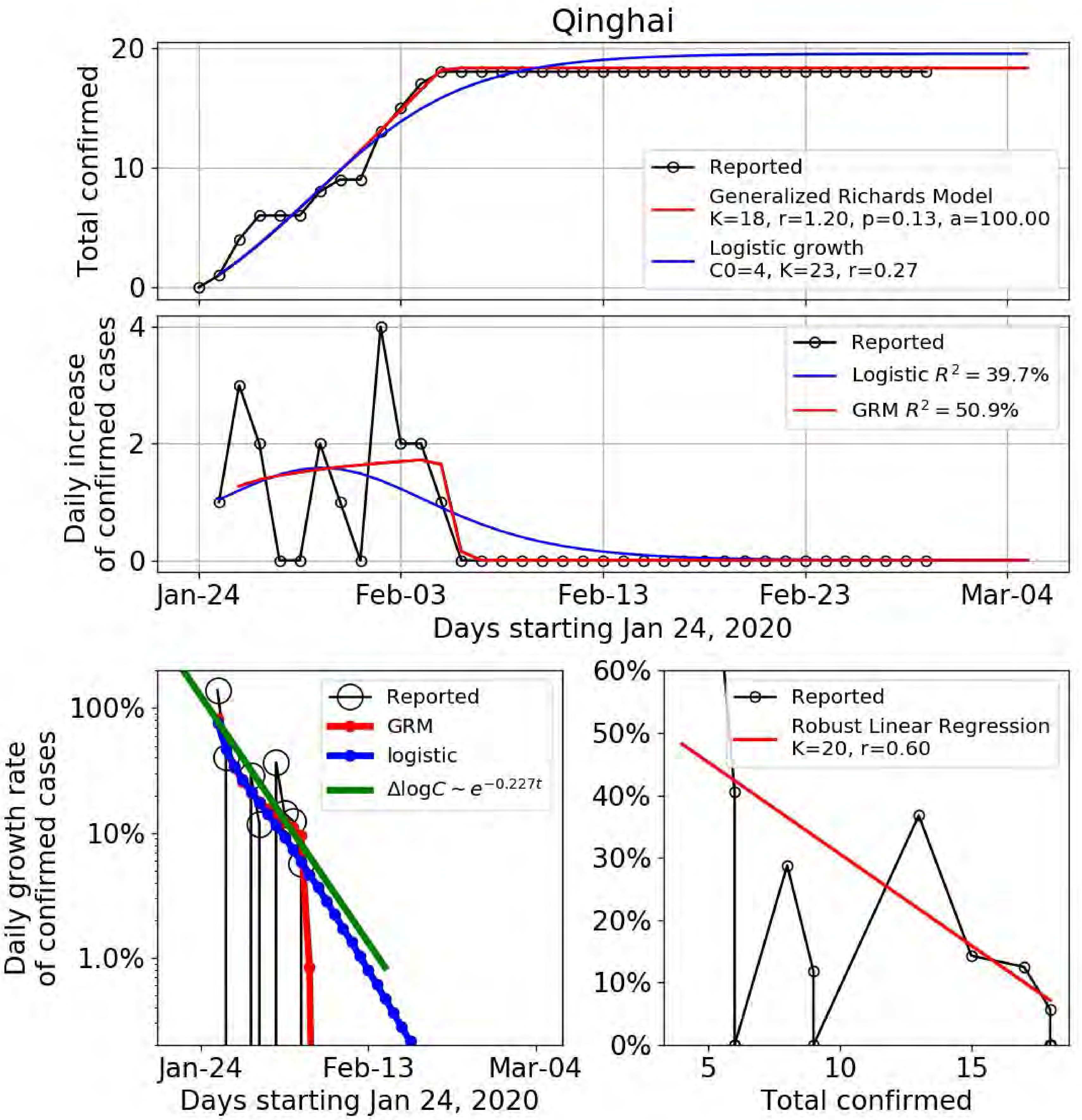

Qinghai
Total confirmed
Reported
Generalized Richards Model
K=18, r=1.20, p=0.13, a=100.00
Logistic growth
C0=4, K=23, r=0.27
Daily increase of confirmed cases
Reported
Logistic $R^2 = 39.7\%$
GRM $R^2 = 50.9\%$
Jan-24
Feb-03
Feb-13
Feb-23
Mar-04
Days starting Jan 24, 2020
Daily growth rate of confirmed cases
Reported
GRM
logistic
$\Delta \log C \sim e^{-0.227t}$
Jan-24
Feb-13
Mar-04
Days starting Jan 24, 2020
Reported
Robust Linear Regression
K=20, r=0.60
Total confirmed
